# HER2 in Non-Small Cell Lung Cancer (NSCLC): Evolution of the Therapeutic Landscape and Emerging Drugs—A Long Way to the Top

**DOI:** 10.3390/molecules30122645

**Published:** 2025-06-18

**Authors:** Pamela Trillo Aliaga, Gianluca Spitaleri, Ilaria Attili, Carla Corvaja, Elena Battaiotto, Panagiotis Agisilaos Angelopoulos, Ester Del Signore, Antonio Passaro, Filippo de Marinis

**Affiliations:** 1 Division of Thoracic Oncology, IEO—European Institute of Oncology, IRCCS, Via Ripamonti 435, 20141 Milan, Italy; 2 Division of New Drugs and Early Drug Development for Innovative Therapies, European Institute of Oncology, IRCCS, Via Ripamonti 435, 20141 Milan, Italy; 3 Department of Oncology and Haematology (DIPO), University of Milan, 20122 Milan, Italy

**Keywords:** NSCLC, adenocarcinoma, HER2, ERBB2, targeted therapy, antibody–drug conjugate, ADC, tyrosine kinase inhibitor, immunotherapy

## Abstract

Non-small-cell lung cancer (NSCLC) can harbour different HER2 alterations: HER2 protein overexpression (2–35%), HER2 gene amplification (2–20%), and gene mutations (1–4%). The discovery of the HER2 gene in the 1980s raised great expectations for the treatment of several tumours. However, it was only in 2004 that HER2 mutations were identified, and they currently represent a key druggable target in NSCLC. Despite numerous strengths, there is only one FDA/EMA-approved targeted therapy, an antibody-drug conjugate (ADC) called trastuzumab deruxtecan for pretreated patients with HER2 mutant NSCLC. In the first-line treatment, the standard of care (SoC) remains chemotherapy with or without immunotherapy. In the past, pan-HER tyrosine kinase inhibitors (TKIs) were extensively studied with poor results. But, two newly developed HER2-specific TKIs with low EGFR WT inhibition (BAY2927088 and zongertinib) reported encouraging results and received the breakthrough therapy designation from the FDA. Ongoing clinical trials are investigating new agents. This review focuses on HER2 alterations. Additionally, the anti-HER2 therapies explored so far will be discussed in detail, including the following: HER2 inhibitors (pan-inhibitors and selective inhibitors), monoclonal antibodies (mAbs), and ADCs. A section of this paper is dedicated to the role of immunotherapy in HER2-altered NSCLC. The last section of this paper focuses on the drugs under development and their challenges.

## 1. Introduction

Non-small cell lung cancer (NSCLC) accounts for approximately 85% of lung cancer cases, representing the leading cause of cancer death worldwide [1]. NSCLC comprises a heterogeneous group of diseases, including adenocarcinoma (45%), squamous cell carcinoma (25%), and other histologies (15%). The use of molecular gene testing platforms has allowed for classifications based on the mutational status of the main driver oncogenes identified, including HER2 mutation. Therefore, NSCLC harbouring HER2 alteration is currently considered a distinct molecular subtype [2].

**Figure 1:**
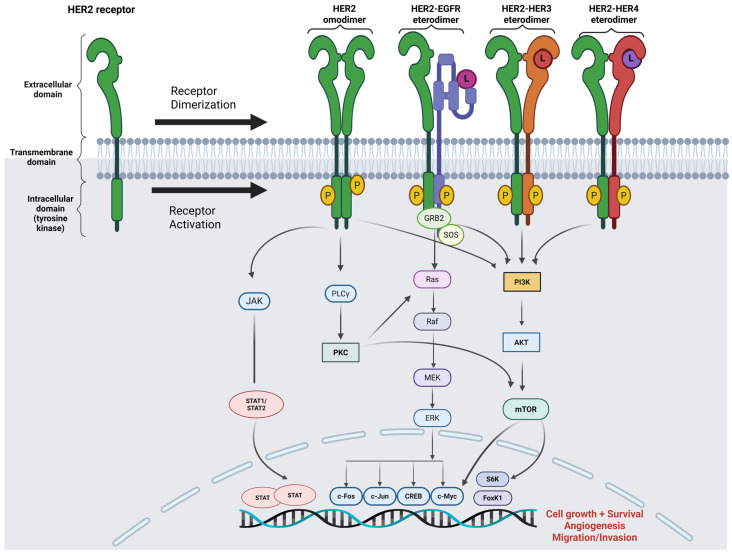
Intracellular molecular pathways due to the activation of the HER2 protein, a transmembrane receptor, after its hetero- or homo-dimerization: MAPK/ERK pathway, PIK3/AKT/mTOR pathway, and JAK/STAT pathway. Abbreviations: EGFR = epidermal growth factor receptor; HER 2/3/4 = human epidermal growth factor receptor-2/3/4; P = phosphate; L = ligand; Ras = rat sarcoma virus proteins; Braf = serine/threonine-protein kinase B-Raf (v-Raf murine sarcoma viral oncogene homolog B); MEK = mitogen-activated protein kinase; ERK = extracellular-signal-regulated kinases; GRB2: growth-factor-receptor-bound protein 2; SOS = son-of-sevenless protein; PIK3 = phosphatidylinositol 3-kinases; AKT = protein kinase B (also called PKB); mTOR = mechanistic/mammalian target of rapamycin. JAK = Janus kinase. STAT = signal transducer and activator of transcription; PLC-γ = phospholipase C-gamma; PKC = Protein Kinase C; c-Fos = cellular oncogene fos; c-Jun = cellular homolog of the viral oncoprotein v-Jun; c-Myc = homology with the viral gene v-myc; CREB = cAMP Response Element-Binding Protein; S6K = Ribosomal protein S6 Kinase; FoXK1 = Forkhead box protein K1. Created using biorender.com.

Human epidermal growth factor receptor 2 (HER2), also known as ErbB2/Neu or cluster of differentiation 340 (CD340), is a transmembrane receptor. HER2 is one of the ErbB family proteins that also include epidermal growth factor receptor (EGFR or HER1/ErbB1), EGFR3 (or HER3/ErbB3), and EGFR4 (or HER4/ErbB4) [3]. The ERBB2 gene is a known proto-oncogene located on chromosome 17, specifically at the 17q11.2-q12 region, which encodes a transmembrane glycoprotein that belongs to the classical superfamily of receptor tyrosine kinases, which have extracellular, transmembrane, and intracellular domains with intrinsic tyrosine kinase activity. Although it is capable of homodimerization, HER2 favours heterodimerization with other ErbB family members (EGFR, HER3, or HER4) when binding to their ligands, but a specific ligand for HER2 has not yet been found [4]. Dimerization of the HER2 receptor activates downstream cascades, including the intracellular pathways of mitogen-activated protein kinases (MAPK), phosphatidylinositol-3-kinase (PI3K)/protein kinase B (AKT), and Janus kinase (JAK)/Signal transducer and activator of transcription (STAT). These signals are indispensable in cell proliferation, migration, and invasion, resulting in the occurrence or progression of malignant tumours (Figure 1).

The ERBB2 gene was first discovered by Weinberg’s group in the early 1980s in rats through experiments on chemically induced neuroblastomas, where its rat homolog “neu” was isolated from the tumour [5]. Subsequently, the discovery of the protein in 1986 generated high expectations for the treatment of different cancers harbouring HER2 aberrations [6,7]. While protein overexpression (OE) and/or gene amplification (AMP) in HER2 has been proved to be important in breast and gastric cancer, in lung carcinogenesis, HER2 mutations (MUT) are thought to be more clinically relevant than HER2 OE or AMP [8], although HER2 MUT account for only 1–4%, while HER2 AMP and HER2 OE is reported to account for around 2–20% and 2–35%, respectively [9,10]. The HER2 MUT was first identified in lung cancer in 2004 [11], and, after its identification as an oncogenic driver in NSCLC, it has been more studied in order to pharmacologically target it.

Regarding the epidemiology of HER2 MUT in lung cancer, small retrospective studies from around the world reported that HER2 mutations are present mostly in adenocarcinoma histology and are more frequent in women, younger patients (median age 60 years old), patients with a low exposure to tobacco (never or light smokers), and in patients of oriental ethnicities, compared to other ethnicities (Table S1) [12,13,14,15,16,17,18,19]. Instead, the characteristics of patients with HER2 AMP or HER2 OE are different and higher-represented in men and smokers, and are not exclusive of adenocarcinoma [20].

Several studies reported that HER2 alterations are associated with poor outcomes. Particularly in two systematic reviews, the poor prognostic role of HER2 AMP/OE was reported. Nakamura et al. included 2579 patients, suggesting significant poorer survival among patients with HER2 OE NSCLC both in resected and advanced settings [21]. Liu et al. included 14 studies with 6135 patients with lung cancer and found that HER2 OE was a marker of poor prognosis [22]. In addition, other retrospective studies confirmed HER2 status to be a poor prognostic factor [18].

Also, brain metastases are associated with a worse prognosis in HER2-altered NSCLC and are present at the onset of advanced disease in 19–30% of cases, and develop during the treatment up to 47% of cases due to the lack of brain activity in classical treatments (chemotherapy and immunotherapy) that do not cross the blood–brain barrier [23,24].

The current first-line standard of care (SoC) for NSCLC harbouring HER2 alterations consists of platinum-based chemotherapy combined with immune-checkpoint inhibitor (ICI) [25]. The overall response rate (ORR) to platinum-based chemotherapy was around 43%, with a median progression-free-survival (mPFS) of around 6 months; in particular, the use of pemetrexed increased the ORRs up to 30–56% in previously untreated patients with HER2-alterated NSCLC [26,27,28]. The therapeutic targeting of HER2 in lung cancer has been studied for decades, and the first targeted therapy, an antibody drug conjugate (ADC) called trastuzumab deruxtecan (T-DXd), was only approved in August 2022 in patients with HER2 MUT who had previously received systemic therapy [29]. However, the optimal targeted therapy for HER2 alterations in NSCLC remains to be determined.

This review will focus on the alterations of HER2 (overexpression, amplification, and mutation) and their methods of determination. Furthermore, we will extensively discuss the anti-HER2 therapies explored so far, including the following: monoclonal antibodies (mAbs), HER2 inhibitors (pan-inhibitors and selective inhibitors), and ADCs. An important section of this paper is dedicated to the role of immunotherapy in NSCLC with HER2 alterations. The last section will focus on drugs currently in development.

## 2. HER2 Alterations

Different types of HER2 alterations have been identified in NSCLC: protein overexpression 2–35%, gene amplification 2–20%, and gene mutation 1–4%. Each HER2 alteration identifies a subgroup with different biological behaviours and which show different responses to anti-HER2 drugs [30,31].

### 2.1. HER2 Overexpression

HER2 OE refers to an abnormally high amount of HER2 proteins on the surface of cancer cells. HER2 expression protein is measured through immunohistochemistry (IHC), with an antibody developed for breast cancer (BC) [32]. HER2 expression measured by IHC can be assessed using different methods, including the ASCO/College of American Pathologist (CAP) BC guidelines [33]. In these guidelines, the final score is 0, 1+, 2+, or 3+ based on membrane staining, among which scores of 0/1+ and 3+ are considered to be negative and positive for HER2 OE, respectively; a score of 2+ is considered equivocal and should be confirmed by additional in situ hybridization (ISH) testing in BC. However, for NSCLC, the guidelines do not recommend ISH confirmation for scores of 2+ because there is not a clear correlation between HER2 amplification and overexpression and this score is considered positive [30].

Unlike breast cancer where HER2 OE subtype disease represents a distinct entity with specific prognosis and has tailored treatments, HER2 OE in NSCLC is not well characterised [34]. In lung cancer, the OE was evaluated in small retrospective studies (ranging from 20 to 563 patients) showing HER2 OE (defined as intermediate or high expression, IHC 2+ or 3+) in 18–35% of patients, with a slightly higher prevalence in adenocarcinomas (28–50%) [35,36,37,38,39,40,41]. Indeed, strong HER2 OE (IHC 3+) seemed to be very low (only 2–6%) [18,32,39,41].

### 2.2. HER2 Amplification

HER2 AMP refers to an increase in the copy number of the HER2 gene, which can occur through focal amplification or polysomy of chromosome 17, where HER2 is located [42]. HER2 AMP can be identified by fluorescent in situ hybridization (FISH) or next-generation sequencing (NGS) in tumour biopsies and/or ctDNA (circulating tumour- deoxyribonucleic acid) [43,44]. The cutoff to arbitrarily define HER2 AMP by FISH is a HER2/CEP17 ratio ≥ 2 [32]. This definition is based on clinical studies for NSCLC and is recommended in guidelines [30]. HER2 AMP assessed in various retrospective trials ranged from 2 to 20% [9,20,45,46]. However, true amplification seems to have a very low incidence (4%) and can be enriched in adenocarcinoma histology [18]. Instead, HER2 polysomy (HER2/CEP17 ratio < 2 with HER2 copy number ≥ 6) has been investigated only in some of these studies, reporting a very wide range of positivity (1.6–53%) [45,46].

In addition to de novo HER2 AMP, an acquired resistance mechanism has been identified in advanced oncogene addicted-NSCLC treated with TKIs, occurring, for example, in approximately 7–15% of cases after post-treatment with EGFR-TKI [47]. HER2 AMP as a mechanism of resistance is well-known; however, it is poorly studied, and it is unknown whether there is sensitivity to anti-HER2 agents because no clinical trials have been conducted in this context to date.

Although the prognostic role of HER2 AMP has been reported, its predictive role in NSCLC remains unclear. Indeed, some reports show that HER2 AMP and HER2 MUT frequently do not overlap in patients and, therefore, may represent distinct clinical entities and different therapeutic targets [13].

### 2.3. HER2 Mutations

As previously mentioned, HER2 mutations have been detected in approximately 1–4% of NSCLC patients [11]. They can be identified using polymerase chain reaction (PCR) or next-generation sequencing (NGS) in either tumour biopsies and/or ctDNA [24].

Oncogenic HER2 mutations are found in both the tyrosine kinase domain (TKD) and non-TKD; consequently, the type of HER2 mutations can affect the tumour’s sensitivity to targeted therapy. The mutations in TKD were first described in 2004 and are the most common [11,34]. They were found in 5 out of 120 (4.2%) NSCLC patients and 5 out of 51 (9.8%) lung adenocarcinoma patients [11]. HER2 mutations are typical almost exclusively of adenocarcinoma, and could be associated with HER2 AMP in a wide range (5–57%) [12,13,14,15,16,17,18,19]. Although HER2 mutations are generally mutually exclusive with other oncogenic drivers, rare co-mutations have been reported. Song et al. described 16 out of 21 cases with concomitant gene mutations: p53 (n = 6), EGFR (n = 3), NF1 (Neurofibromatosis type 1 gene) (n = 3), KRAS (Kirsten Rat Sarcoma viral oncogene homolog) (n = 2), and others (n = 2) [17].

**Figure 2:**
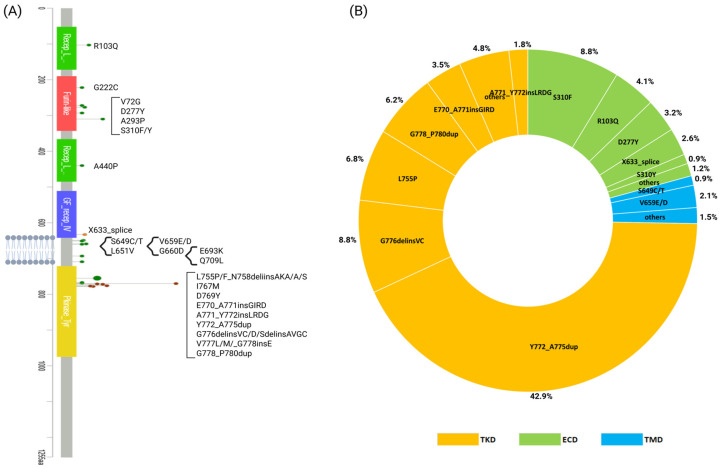
HER2 mutations (considering only in oncogenic and likely oncogenic mutations) reported in 35 NSCLC studies in a large public database (cbioportal). (**A**) Representation of these mutations in the tyrosine-kinase domain (TKD), extracellular domain (ECD), and transmembrane domain (TMD). (**B**) Graphical representation of the frequency of the most representative mutations for each HER2 domain (TKD, ECD, TMD). Abbreviations: TKD: tyrosine-kinase domain, ECD = extracellular domain, TMD = transmembrane domain. Created using biorender.com.

Overall, from 35 NSCLC studies in the public database (cbioportal), we only considered oncogenic and likely oncogenic HER2 mutations (Table S2) (Figure 2) [48].

HER2 mutations in TKD have a prevalence of around 74%. These mutations occur most commonly in exon 20 (~90%), followed by exon 19 (~6%), exon 18 (~4%), and exon 21 (<1%) [49,50]. The most frequent is A775_G776insYVMA (alternatively called p.772_A775dup [c.2313_2324dup]) (~43%), which is a 12 bp duplication/insertion of the amino acid sequence YVMA in exon 20 (Ex20) at codon 776. Other Ex20 insertions include G776delinsVC (~9%) and G778_P780insGSP (~6%). The insertion in Ex20 of the HER2 gene results in a conformational change in the TKD that leads to a narrowing of the ATP-binding cleft and, consequently, to an increase in kinase activity compared to the wild-type HER2 receptor (HER2 WT) [8]. Also, it is speculated that these mutations confer intrinsic resistance to pan-HER TKIs because they result in the steric hindrance of the drug-binding pocket [51].

The second most frequent mutations are the HER2 mutations in the extracellular domain (ECD) (~21%); the most recurrent are S310F (8.8%), D277Y (3.2%), and X633_splice (2.6%). Many reports document that mutations in the ECD of the HER2 are oncogenic and are associated with sensitivity to treatment with HER2 inhibitors (Table S2) [52].

The next most frequent mutations are HER2 mutations in the transmembrane domain (TMD) (~5%), and the most representative is V659E (1.8%). Finally, some reports also report very rare HER2 mutations in the intracellular domain, which includes exons 22–31, and the most representative is E1021Q [34].

## 3. Targeting HER2

This section aims to review the knowledge and studies targeting HER2-positive NSCLC. In the last decade, the treatment of HER2-positive NSCLC has been arduously investigated. However, the current standard first-line therapy is represented from platinum-based chemotherapy combined with ICI, and, despite numerous efforts, no first-line targeted therapy has been identified to date [25].

### 3.1. Monoclonal Antibodies

Monoclonal antibodies (mAbs) have been extensively explored in HER2-altered NSCLC. Clinical trials of the anti-HER2 mAb trastuzumab alone or in combination with chemotherapy have shown negative results in HER2 OE NSCLC, while some activity has been observed in HER2 AMP NSCLC (Table 1) [53,54,55,56,57,58,59,60,61].

In 2018, Kinoshita et al. conducted a trial of trastuzumab monotherapy in ten pretreated HER2-positive lung adenocarcinoma patients (three with HER2 OE, and seven with HER2 MUT), observing no objective responses. However, 70% achieved stable disease, with a median PFS of 5.2 months [53]. The addition of chemotherapy (first-line gemcitabine-cisplatin or first-/second-line docetaxel/paclitaxel) to trastuzumab in five different trials failed to demonstrate a benefit in HER2 OE patients with ORRs that ranged from 23 to 36% in first-line and 8–24% in second-line treatment and beyond [54,55,56,57,58].

The combination of trastuzumab and pertuzumab alone or in combination with docetaxel also did not report encouraging results, with ORRs of 8–21% in dual blockade alone and an ORR of 29% in dual blockade plus docetaxel in pretreated NSCLC patients with HER2 AMP or MUT [59,60,61].

These trials are summarised in Table 1. Despite the numerous limitations of these studies, such as their non-randomised phase II nature, small sample size of patients (ranging from 10 to 101), and heterogeneous eligibility criteria—where some trials enrolled unselected patients and others only patients with HER2 OE or AMP or MUT (not all trials indicated exactly which ones)—taken together, the results of trastuzumab- +/− pertuzumab-based combinations fail to show activity, even in patients with HER2 MUT [61].

### 3.2. Tyrosine-Kinase Inhibitors

TKIs are small molecules directed against the tyrosine kinase enzymes in the intracellular domain of the HER2 receptor. In this section, we summarise the principal trials on HER2-directed TKIs, dividing them into three main categories: pan-HER TKIs (dacomitinib, neratinib, afatinib, poziotinib, tarloxotinib), EGFR/HER2 TKIs (lapatinib, pyrotinib mobocertinib, BAY2927088), and selective HER2 TKIs (tucatinib, zongertinib). This classification follows a single criterion based on the specificity of the HER2 inhibition: against EGFR/HER2/HER4 for pan-HER TKIs, against EGFR/HER2 for dual TKIs, and against HER2 for selective TKIs (Table 2).

#### 3.2.1. Pan-HER TKI

Initial efforts to target HER2 alterations in NSCLC with pan-HER inhibitors resulted in disappointing outcomes. Table 2 summarises the results of clinical trials with pan-HER inhibitors. Overall ORRs ranged from 0 to 42% and mPFS ranged from 2.8 to 5.6 months.

Dacomitinib is an irreversible pan-HER TKI. It was studied in a phase II trial in 30 patients that were HER2-positive (26 MUT, 4 AMP), with limited results in the HER2 MUT subgroup: ORR 12% (two patients with Ex20 insertion), mPFS 3 months, and mOS 9 months; with scarce tolerability, the most commons adverse events (AEs) reported were diarrhoea at 90% (grade 3 or greater [G ≥ 3] 23%) and skin rash at 73% (G ≥ 3 3%) [62].

Neratinib, as a monotherapy or combined with either temsirolimus (mTOR inhibitor) or trastuzumab (mAb), also has limited activity in HER2 MUT NSCLC, with ORRs that ranged 0–8%, mPFS 2.9–5.4 months, and mOS 10–15.8 months in two studies that included both naïve and pre-treated patients; the tolerability was similar to other pan-HER TKI, and the most common AEs were diarrhoea, stomatitis, constipation, nausea, and vomiting [63,64].

Afatinib is another irreversible pan-HER TKI that did not obtain great efficacy (ORR 7%, mPFS 15.9 weeks, and mOS 56 weeks) in the NICHE phase II trial that enrolled 13 pre-treated patients with HER2 MUT (Ex20) NSCLC [65], another phase II trial did not report efficacy [66], and the most common AEs were diarrhoea, skin rash, mucositis, and transaminitis [65,66].

Poziotinib is another irreversible pan-HER TKI [49,67]. In cohort 2 of the ZENITH20 phase II trial, 90 patients with HER2 MUT NSCLC received poziotinib 16 mg once daily and achieved an ORR of 27.8%, disease control rate (DCR) of 70%, and mPFS of 5.5 months; however, significant toxicities were reported, including skin rash 83% (G ≥ 3 47%), diarrhoea 80% (G ≥ 3 20%), and stomatitis 67% (G ≥ 3 10%), which led to a 13.3% discontinuation rate [67]. Cohort 4 of the ZENITH20 trial enrolled 80 naïve patients with HER2 MUT NSCLC treated with 16 mg once daily or 8 mg twice daily (alternative schedule to try to reduce the toxicity). Nevertheless, rates in G ≥ 3 AEs did not differ in the two dose arms (around 45%). The ORR was 39% and mPFS was 5.6 months in the overall patient sample with both schedules. In this trial, patients with the G778_P780dupGSP mutation (9%) had a higher ORR (88%) [68]. Significant toxicity (skin rash and diarrhoea) was also reported in another phase II study [69]. However, a phase 3 study (PINNACLE) comparing poziotinib versus (vs) docetaxel in pretreated HER2 MUT (Ex20) NSCLC was prematurely withdrawn due to the company’s decision not to fund the study, likely because the safety results from the early-phase trials were not convincing, despite the promising emerging activity [79].

tarloxotinib is a prodrug and generated a potent pan-HER TKI (tarloxotinib-E) using hypoxia in the tumour microenvironment [80]. In cohort B of a phase II trial, 11 pretreated patients with HER2 MUT NSCLC treated with this drug reported an ORR of 22% and a DCR of 66%; regarding safety, the most common AEs were prolonged QTc (corrected QT interval) at 60.9% (G ≥ 3 34.8%), skin rash at 43.5% (G ≥ 3 4.3%), and diarrhoea at 21.7% G ≥ 3 4.3%) [70].

#### 3.2.2. EGFR/HER2 TKI

Considering that HER2-alterated NSCLC may benefit from the dual inhibition of EGFR and HER2, TKIs that simultaneously targeting EGFR/HER2 have been investigated.

The first was lapatinib, an oral reversible TKI against EGFR and HER2, that, in a phase II trial, did not report responses in HER2 MUT NSCLC [71], so its investigation was withdrawn for lung cancer due to the lack of efficacy (NCT00073008).

Pyrotinib is an irreversible TKI targeting both HER2 and EGFR, and it has shown promising activity. In two Chinese phase II trials in pre-treated HER2 MUT (Ex20/others) NSCLC patients treated with pyrotinib, ORRs ranged from 19 to 53%, mPFS ranged from 5.6 to 6.4 months, and mOS ranged from 10.5 to 12.9 months [72,73]. Additionally, pyrotinib was studied in a first-line setting in 48 patients with HER2 MUT NSCLC. In the criteria fulfilled (CF) cohort (n = 28) and compassionate use (CU) cohort (n = 12), the ORRs were 35.7% and 16.7%, DCR were 89.3% and 83.4%, mPFS were 7.3 and 4.7 months, and mOS were 14.3 and 14.2 months, respectively [74]. However, diarrhoea, anaemia, and skin rashes limited its tolerability [72,73,74]. Therefore, pyrotinib is currently being evaluated in a phase 3 PYRAMID-1 study, which compares it with docetaxel in patients with advanced HER2 MUT (Ex20) NSCLC after platinum-based chemotherapy (NCT04447118).

Mobocertinib (TAK-788) is another potent irreversible TKI designed to target EGFR/ERBB2 Ex20 insertions that showed interesting activity in pre-clinical models [81]. Recently, Kanemura et al. reported an ORR of 53.8%, a DCR of 84.6%, and mPFS 6.1 months in 13 pretreated HER2 MUT NSCLC patients treated with mobocertinib and TDM1, including those who had developed resistance to T-DXd [75].

BAY2927088 (sevabertinib) is a novel oral reversible TKI designed to target both HER2 (Ex20 insertions and point mutations) and EGFR mutations. In the cohort D of the SOHO-01 phase I/II trial, which included 44 patients with HER2 MUT (Ex20) NSCLC who were pretreated but naïve for anti-HER2 therapies, BAY2927088 achieved an ORR of 72.1%, a DCR 96%, and a mPFS of 7.5 months. Greater efficacy is reported in patients with A775_G776insYVMA (ORR of 90%, DCR of 97%, and mPFS of 9.9 months). Also, patients with brain metastasis (n = 8) derived benefits. The treatment was well tolerated, and the most common AEs were diarrhoea 86.4% (G ≥ 3 25%), skin rash 43% (all G1-2), and paronychia 25% (all G1-2). Only 6.8% of drug discontinuation and 31.8% of dose reduction was reported [76]. In light of these promising results, BAY2927088 received the breakthrough therapy designation (BTD) from the Food and Drug Administration (FDA) for the treatment of patients with advanced NSCLC with activating HER2 mutations [82]. Interestingly, at the last European Lung Cancer Conference (ELCC 2025), Girard et al. reported an update to this trial, showing that BAY2927088 attained an ORR of 70.5% and mDOR of 8.7 months in the cohort D (44 patients with HER2 MUT NSCLC naïve to anti-HER2 ADCs) and an ORR of 35.3% with a mDOR of 9.5 months in the cohort E (34 patients with HER2 MUT NSCLC previously treated with anti-HER2 ADCs) [83]. Furthermore, recently, at the last American Society of Clinical Oncology (ASCO) annual meeting, the preliminary analysis of cohort F (39 patients with HER2 MUT NSCLC naïve for systemic therapy) was presented with an ORR of 59% and a DCR of 84.6% [84]. BAY2927088 is currently being investigated in a randomised phase III trial in first-line HER2 MUT (TKD mutations) NSCLC patients that compared BAY2927088 vs. SoC (cisplatin/carboplatin + pemetrexed + pembrolizumab), and the primary endpoint is PFS, as measured by a blinded independent central review (BICR) (SOHO-02, NCT06452277). In the meantime, the Expanded Access Program is open to allow patients with advanced HER2 MUT NSCLC, without other treatment options, to receive BAY2927088 (NCT06761976).

#### 3.2.3. Selective HER2 TKI

Selective HER2 TKIs are designed to selectively inhibit the HER2 receptor, with minimal impact on other members of the EGFR/ERBB family. These drugs, which attempt to spare wild-type EGFR (EGFR WT) inhibition, could help improve their safety profiles.

Tucatinib is an oral reversible TKI highly selective only for HER2, and it is being investigated in association with trastuzumab in a phase II study for all solid tumours that includes HER2-alterated (AMP and MUT) NSCLC (SGNTUC-019 trial) [77]. The results of this study have not yet been published.

Zongertinib (BI 18106319) is a novel irreversible HER2-specific TKI. Recently, the primary analysis of a Beamion LUNG-1 study was published, a phase Ia/Ib multicohort trial in previously treated patients that received zongertinib [78,85]. In particular, the last update reported the results of cohort 1 (patients with HER2 MUT in TKD), cohort 3 (patients with HER2 MUT with non-TKD), and cohort 5 (patients with HER2 MUT in TKD and previously treated with ADC). In cohort 1, 75 patients were enrolled, and the ORR was 71% (95% CI, 60–80), mDOR was 14.1 months (95% CI, 6.9-NE), and mPFS was 12.4 months (95% CI, 8.2-NE). In cohort 3, 20 patients were enrolled, and the ORR was 30% (95% CI, 15–52). In cohort 5, 31 patients were treated with zongertinib after ADC, and the ORR was 48% (95% CI, 32–65). Patients with brain metastasis numbered 28 in cohort 1 and obtained an ORR of 65%. The drug was very well tolerated, with a low incidence of dose reductions (7% in cohort 1) and treatment discontinuations (3% in cohort 1). G ≥ 3 occurred in 17%, 25%, and 3% in cohort 1, 3, and 5, respectively. No cases of drug-related interstitial lung disease (ILD) occurred in all three cohorts [78]. In light of these impressive data, in August 2024, zongertinib received BTD from the FDA for the treatment of adult patients with advanced HER2 MUT NSCLC [86]. Currently, a phase III trial that compared zongertinib (BI 1810631) to the SoC (platinum + pemetrexed + pembrolizumab) is ongoing in previously untreated patients with HER2 MUT (in TKD) NSCLC, and the primary endpoint is BICR-PFS (Beamion LUNG-2, NCT06151574).

**Figure 3:**
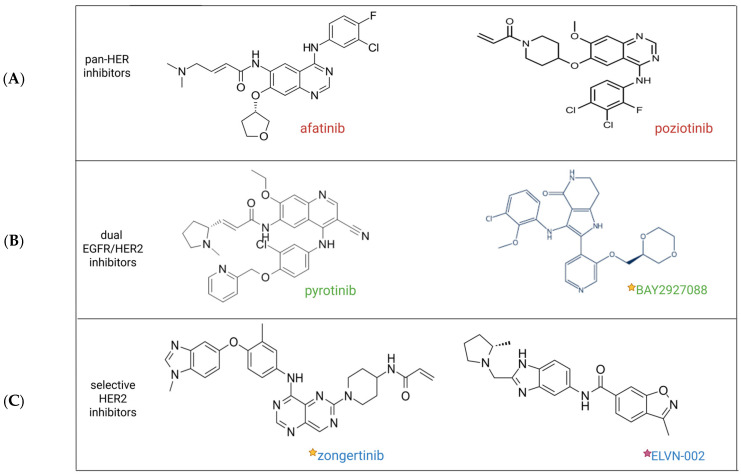
Illustration of type of HER2 tyrosine-kinase inhibitors (TKIs) explored in HER2-mutated NSCLC. (**A**) Two of various pan-HER TKIs (afatinib and poziotinib). (**B**) Two of many dual EGFR/HER2 TKIs: pyrotinib and BAY2927088. (**C**) Two new selective HER2 TKIs: zongertinib and ELVN-002. Legend: yellow stars: promising drugs for which phase I/II data are published; purple star: drug for which phase I trial is ongoing. Abbreviations: TKIs: tyrosine-kinase inhibitors. ELVN: Eleven-Nineteen-Leukemia Protein IN-2. Created using biorender.com.

New promising HER2-selective TKIs are under investigation, such as ELVN-002, which we will discuss later in a dedicated section. Figure 3 illustrates the chemical structure of the three categories of TKIs, providing some examples. Furthermore, Table S3 summarises the characteristics of these drugs.

### 3.3. Antibody–Drug Conjugates

ADCs are novel therapeutic agents composed of mAb carrying a cytotoxic drug (payload) through a linker [87]. These drugs were developed to improve the therapeutic window of conventional chemotherapy through selective delivery to tumour cells expressing the target antigen, thereby limiting the potential off-target systemic toxicities [88]. Several ADCs are currently under investigation for lung cancer; here, we only describe the anti-HER2 ones [89] (Table 3).

**Figure 4:**
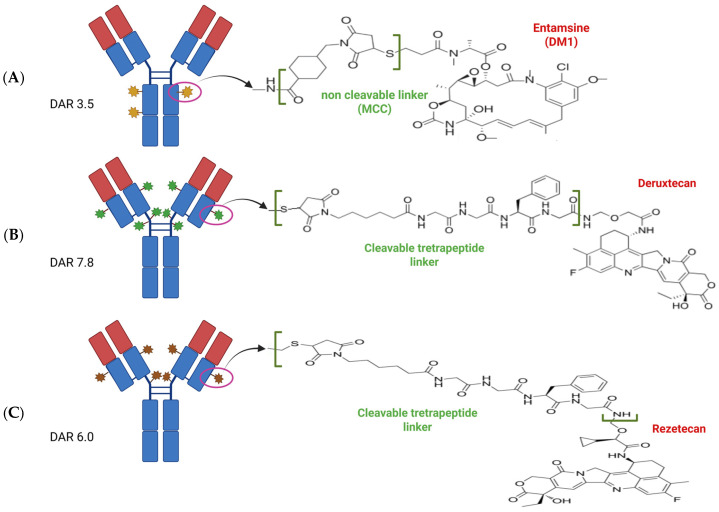
Illustration of antibody–drug conjugates (ADCs) investigated in HER2-altered NSCLC. (**A**) Trastuzumab emtamsine (TDM1), a second-generation ADC. (**B**) Trastuzumab deruxtecan (TDXd), a third-generation ADC (**C**) Trastuzumab rezetecan (SHR-A 1811), another third-generation ADC. Created using biorender.com.

Ado-trastuzumab emtansine (T-DM1) is a HER2-targeted ADC that links trastuzumab to the anti-microtubule agent emtansine (Figure 4, Table S4). Several phase II studies have evaluated the activity of T-DM1 in HER2-alterated NSCLC with modest results, with ORRs ranging from 0 to 20% and from 14.3 to 50% in OE HER2- and MUT HER2 NSCLC, respectively [90,91,92,93].

The most promising data come from trastuzumab–deruxtecan (T-DXd or DS-8201), a HER2-targeted ADC that links trastuzumab to a topoisomerase I inhibitor payload via a tetrapeptide-based cleavable linker (Figure 4, Table S4). A phase I trial in all solid tumours reported a good safety profile for T-DXd and interesting results in NSCLC patients [94]. The clinical benefit of T-DXd at doses of 6.4 mg/kg was demonstrated in a phase II DESTINY-Lung01 trial (cohort 2 including 91 patients with HER2 MUT NSCLC), which reported an ORR of 54.9%, mPFS of 8.2 months (95% CI, 6.0–11.9), and mOS of 17.8 months (95% CI, 13.8–22.1). The 33 patients with brain metastases (stable and asymptomatic at baseline) also achieved the following benefits: ORRs of 54.5%, mPFS of 7.1 months, and mOS of 13.8 months. Regarding safety, G ≥ 3 AEs were 46%, and the most common was neutropenia (19%). The incidence of drug-related ILD was 27.5%, with two fatal cases. Dose interruption, dose reduction, and discontinuation rates were 32%, 34%, 25%, respectively [95]. A subsequent publication reported patients with HER2 OE (IHC 2+ and IHC 3+), cohort 1 (49 patients treated with T-DXd at 6.4 mg/kg), and cohort 1A (41 patients treated with T-DXd at 5.4 mg/kg), with ORRs of 26.5% and 34.1%, mPFS of 5.7 months and 6.7 months, and mOS of 12.4 months and 11.4 months in cohort 1 and cohort 1A, respectively. In a post hoc analysis, antitumor activity was also observed in patients with HER2 AMP (13 patients in A and 9 patients in 1A) and in patients who had received prior TKIs (17 patients in A and 7 patients 1A). AEs of G ≥ 3 were 53% and 22% in cohort 1 and cohort 1A, respectively. Instead, ILD occurred in 20% (G ≥ 3 6%) and 5% (G ≥ 3 2%) in cohort 1 and cohort 1A, respectively [96]. DESTINY-Lung02 is a phase II study that evaluated the efficacy and safety of T-DXd 5.4 mg/kg and 6.4 mg/kg in 102 and 50 patients with HER2 MUT NSCLC, respectively, obtaining very interesting results: ORRs of 50% and 56%, mPFS of 10.0 months and 12.9 months, and mOS of 19.0 months and 17.3 months with 5.4 mg/kg and 6.4 mg/kg, respectively. AEs of G ≥ 3 were 39% and 60% with 5.4 mg/kg and 6.4 mg/kg, respectively. ILD was 14% (G ≥ 3 2%) and 32% (G ≥ 3 2%) at the two doses, respectively [97,98]. Based on the results of these trials in 2024, T-DXd was the first target therapy approved by the FDA for patients with previously treated HER2 MUT NSCLC at a dose of 5.4 mg/kg [100]. Currently, a phase II study evaluating T-DXd in a Chinese population after at least one line of treatment is ongoing (DESTINY-Lung05, NCT05246514). Also, a phase II trial (ELPIS) is ongoing to evaluate the efficacy and safety of T-DXd in HER2 MUT NSCLC with asymptomatic brain metastases, with the primary endpoint being intracranial-progression-free-survival (IC-PFS), as defined by Response Assessment in Neuro-Oncology (RANO) criteria (NCT06250777). Finally, the DESTINY-Lung04 is ongoing—a global, open-label, randomised, phase III study evaluating T-DXd vs. SoC (platinum+ pemetrexed+ pembrolizumab) as first-line treatment in patients with advanced NSCLC harbouring HER2 MUT (Ex 19 or 20) (NCT05048797).

Trastuzumab rezetecan (SHR-A1811) is a novel ADC consisting of a mAb, a cleavable tetrapeptide linker, and a DNA topoisomerase I inhibitor (Figure 4, Table S4). In the phase II HORIZON-Lung study conducted in China, 94 pretreated patients with HER2 MUT NSCLC achieved an ORR of 73% and a mPFS of 11.5 months; OS data was not mature. Activity was evident in various subgroups: in 24 patients with stable brain metastases (ORR of 87.5% and mPFS of 9.9 months), in 22 patients previously treated with pan-HER TKIs (ORR of 77.3%), and in patients with various levels of HER2 expression (ORR: 70%, 75%, 83% for IHC score of 0, 1+, 2+, respectively). AEs of G ≥ 3 were 62% (the most common were neutropenia 40%, leukopenia 27%, anaemia 23%, and thrombocytopenia 11%). ILD was 7% (G3 1%). Treatment-related AEs (TRAEs) leading to dose interruption, dose reduction, and discontinuation occurred in 30%, 17%, and 1% of cases, respectively [99]. Currently, a phase III trial comparing SHR-A1811 vs. SoC (camrelizumab + pemetrexed/paclitaxel + carboplatin/cisplatin) in first-line HER2 MUT NSCLC is ongoing in China (NCT06430437).

## 4. HER2 and Immunotherapy

Pivotal clinical trials of ICI did not include patients with known HER2-alterated NSCLC; therefore, our knowledge of efficacy in this target patient population can only be derived from translational research and retrospective trials (Table 4) [101,102,103,104,105,106,107,108,109,110,111,112].

Previous reports demonstrated that HER2 MUT NSCLC had low tumour mutational burden (TMB) and low or negative PD-L1 expression [107,110]. In particular, PD-L1 expression in HER2 MUT NSCLC was lower than in unselected NSCLC, and TMB was similar to unselected NSCLC (median TMB 5.4 mutations/megabasis [Mt/Mb] vs. 5.7 Mt/Mb) [102,103].

Retrospective analyses reported low response rates to single-agent ICI in patients with HER2 MUT or AMP NSCLC, with ORRs of 6–29% and 0–12%, respectively. In patients with HER2 MUT NSCLC, responses were correlated with high PD-L1 or high TMB [102,107]. Instead, in patients with HER2 AMP NSCLC, no correlations with these biomarkers were found [101,109]. However, these retrospective studies have several limitations, such as the small number of patients, differences in baseline characteristics (smoking status, PD-L1 expression, type of HER2 alteration), and the number and type of previous therapy, thus precluding definitive conclusions.

Dudnik et al. reported on 14 patients with HER2-positive (9 MUT and 5 AMP) NSCLC treated with ICI. The activity of ICI was modest in both groups of patients: ORR 20% vs. 12%; mPFS 3.4 months vs. 6.3 months; and mOS 17.5 months vs. 10.4 months, respectively [101]. Similarly, DeMatteo et al. did not report any responses in 18 patients with HER2 AMP NSCLC [109].

The IMMUNOTARGET registry was a large European multi-centre retrospective trial involving patients with oncogenic driver alterations that enrolled 551 patients with NSCLC. ICI was given mostly as the second- (41%) or third-line (26%) setting. In total, 29 patients had HER2 MUT (Ex20) NSCLC; of these, 27 patients had PD-L1 score (57% with negative score, 53% with 1–49% and 0% ≥ 50%), and 14 (51.9%) patients were never-smokers. In this analysis, the ORR was 7.5%, the mPFS (principal endpoint of the study) was 2.5 months, and the OS was 21.3 months. In the univariate analysis, PFS significantly correlated with smoking status and PD-L1-positive status [104].

Two studies investigating the efficacy of ICI in HER2 MUT NSCLC had a high rate of unknown or missing PD-L1 status (65–82%). The first study (GFPC01-2018) enrolled 23 patients (65% never smokers) and showed an ORR of 27.3%, mPFS of 2.2 months, and mOS of 20.4 months. Interestingly, predictive factors for response to ICI were treatment in early lines and PD-L1 positivity among patients with PD-L1 availability [105]. In the other study, 50 patients were included in two datasets (MDACC and CGDB); again, despite the limitations of biomarker availability, the authors found a positive association between PFS and OS with positive PD-L1 (both datasets) and TMB >10 Mt/Mb (only for CGDB dataset) [107].

Lau et al. also analysed 14 patients with HER2 MUT NSCLC, reporting an ORR of 29% and mPFS of 3.6 months. They also compared in-frame insertions with missense substitutions in HER2 subgroups and did not find a differential effect of the type of mutation to ICI response. Moreover, they also showed that the presence of co-mutations (e.g., TP53, STK11, MSH6) did not correlate with the PFS [106].

The low efficacy of ICI alone for HER2-altered NSCLC might be attributed to three main mechanisms: (1) “cold phenotype” due to a low cytotoxic CD8^+^ T cell infiltration and PD-L1 expression; (2) low TMB, which could be associated with low tumour-specific antigens; (3) and a lack of co-mutations, which makes the tumour more sensitive to immunotherapy [109]. Tian et al. reported that other mechanisms, such as DNA damage repair (DDR) pathways and mammalian SWItch/Sucrose Nonfermentable (SWI/SNF) chromatin remodelling complexes (es. ARID1A and ARID1B), might contribute to responses to ICI [110].

Instead, retrospective data of first-line chemoimmunotherapy demonstrated ORRs of 29–60% and mPFS of 5–8 months in HER2 MUT NSCLC patients, similar of those attained in non-oncogene-addicted NSCLC [108,111,112,113]. Perhaps chemotherapy may stimulate the release of tumour antigens and potentially upregulate the expression of major histocompatibility complex (MHC) class I molecules, which could enhance tumour antigen presentation and thus improve responses to ICI [114].

In summary, although responses to single ICI therapy are lower in heavily pretreated HER2 MUT NSCLC patients, we can see that many reports demonstrate a positive association between positive/high PD-L1, high TMB, and smoking habits. In fact, the use of first-line chemo-immunotherapy remains the recommended SoC, and ICI should not be avoided, especially if the patient presents these characteristics.

## 5. New Drugs in Development

Table S5 collects the drugs in development and the strategies to treat the patients with NSCLC-harbouring HER2 alterations. Below, we summarise, in subsections, the most important and promising categories.

### 5.1. New HER2-Directed TKIs

Regarding the new TKIs, in addition to improving their efficacy, toxicity remains the challenge to overcome. In this sense, the development of specific HER2-TKIs with low inhibition of EGFR WT could improve their efficacy and tolerability. Promising new selective-TKIs under investigation are NVL-330 (HEROEX-1, NCT06521554), ELVN-002 (NCT05650879), and IAM1363-01 (NCT06253871); other TKIs active both for HER2/EGFR under investigation are HS-10376 (NCT05435274) and BH-30643 (SOLARA, NCT06706076) in NSCLC; and ABT-101 (NCT05532696), YH42946 (NCT06616766), ORIC-114 (NCT05315700), and TAS2940 (NCT04982926) in all solid tumours, which also include the HER2-altered NSCLC. Moreover, some of these trials are evaluating the efficacy of these TKIs toward brain metastases, including the following as primary or secondary endpoints: NVL-330 (HEROEX-1), IAM1363-01 (NCT06253871), and ORIC-114 (NCT05315700).

### 5.2. New HER2-Directed Bispecific Antibodies

Zanidatamab (ZW25), instead, is a humanised bispecific monoclonal antibody (bsAb) directed against two domains of HER2: the juxtamembranous domain (ECD4) and the dimerization domain (ECD2). In the first-in-human phase I dose-escalation and expansion, ZW25 was evaluated in 46 patients (part 1) and 86 patients (part 2) with HER2 OE or HER2 AMP solid tumours (including NSCLC); the RDP2 was 20 mg/kg every 2 weeks, and the most common treatment-related AEs were diarrhoea (43%) and infusion reaction (34%), with only 3% of G3-4 of AEs. In part 2, the ORR was 37% (95% CI 27–49), median DOR was 8·5 months (95% CI 3·2-NE), and mPFS was 5·4 months (95% CI 3·7–7·3) [115].

### 5.3. New Anti-HER2 ADCs

Another promising class of drugs are the new ADCs; the most promising among these is Disitamab vedotin (DV or RC48), which contains three components: a humanised IgG1 monoclonal antibody directed to HER2, a microtubule-disrupting agent MMAE (monomethyl auristatin E), and a protease-cleavable mc-vc (maleimidocaproyl-valine-citrulline) linker that covalently attaches MMAE to the antibody. Importantly, the drug gained fast approval from the Chinese National Medical Products Administration (NMPA) for patients with HER 2 OE (score 2+/3+) gastric cancer who had previously received at least two lines of chemotherapy and for pretreated patients with HER2 OE (score 2+/3+) urothelial carcinoma [116,117]. Therefore, RC48 as a monotherapy (NCT04311034, NCT06003231) or in combination is also in clinical development for the treatment of other solid tumours, including NSCLC.

Other novel ADCs under investigation in NSCLC in phase I/II trials are BL-M17D1 (NCT06114511), TQB2102 (NCT06496490), and MRG002(NCT05141786); and more new ADCs under evaluation in all solid tumours, including NSCLC, in phase I trials are DB-1303/BNT323 (NCT05150691), XMT-2056 (NCT05514717), and GQ1001 (NCT04450732).

### 5.4. Combination Therapies

Since T-DXd became the first approved targeted therapy, strategies to improve its efficacy include combinations with other agents, including immunotherapy. In the umbrella (HUDSON) trial in previously treated patients with chemoimmunotherapy, the combinations of T-DXd + durvalumab (anti-PD-L1) reported ORRs of 26.1% and 35%, mPFS of 2.8 and 5.7 months, and mOS 9.5 and 10.6 moths in HER2 OE (n = 23) and HER2 MUT (n = 20) patients, respectively. All grades of ILD were 9.3% (G ≥ 3 7%) in HER2 OE patients, and 10% (G ≥ 3 5%) in HER2 MUT patients [118]. The phase IB trial (U106 trial, NCT04042701) explored the combination of T-DXd and pembrolizumab in naïve or previously treated patients with HER2 OE or MUT NSCLC. In the HER2 OE NSCLC pretreated patients (n = 22), the ORR was 54.5%, mDoR was 20.2 months, and mPFS was 15.1 months; in the naive patients (n = 8), ORR was 62.5%, mDoR was 20.2 months, and mPFS was 23.5 months. In the HER2 MUT NSCLC (n = 33), ORR was 66.7%, mDoR was 15.1 months, and mPFS was 11.3 months; in the naïve patients (n = 20), the ORR was 80%, mDOR was 19.9 months, and mPFS was 21.3 months. ILD remained an important risk with this treatment: G ≥ 2 were 9–27% in both cohorts [119]. Similarly, DESTINIY-Lung06 is an ongoing phase III, randomised, open label trial that compares T-DXd plus pembrolizumab vs. SoC (platinum/pemetrexed/pembrolizumab) in naïve patients with HER2-OE with PD-L1 < 50% (NCT06899126). Finally, DESTINY-Lung03 part 4 is phase Ib study combining T-DXd plus rilvegostomig (anti-PD1 and anti -TIGIT bsAb) ± carboplatin as a first-line treatment for advanced HER2 OE NSCLC [120].

### 5.5. New Modalities (Novel Immunotherapies, Cell Therapies, and Nanotherapies)

BDC -1001 is an immune stimulating antibody conjugate (ISAC) that combines a tumour-targeting antibody with an immune-stimulating payload, and it is being investigated in a phase I/II trial in combination with nivolumab in all solid tumours with HER2 OE or AMP, including NSCLC (NCT04278144). Instead, DF1001 is the first-in-class, Tri-specific, Natural Kille (NK) cell Engager Therapy (TriNKET) which uses HER2 as an anchor to modulate innate and adaptive immunity, and it is being studied in phase I/II in all solid tumours, including NSCLC with HER2 alteration (MUT, AMP, and OE) (NCT04143711).

ACE1702 (anti-HER2 NK cells) and anti-HER2-CAR [chimeric antigen receptor]-macrophages are cell therapies being investigated in first-in-human phase I clinical trials in all solid tumours, including NSCLC with HER2 OE (NCT04319757, NCT04660929). Also, the HER-2/Neu vaccine, improved with new post-pandemic technology, is being developed in solid tumours with HER2 OE (NCT00003002).

Finally, ABP-102/CT-P72, a tetravalent bispecific HER2xCD3 T-cell engager, demonstrates, in preclinical data, potent and highly selective tumour-killing activity toward HER2 OE tumours [121]. Furthermore, emerging nanomedicine approaches in targeted lung cancer treatment represent an innovative strategy, offering novel nanomaterials with the potential to precisely target tumour cells while sparing healthy tissues. Ongoing studies are exploring the use of different nanoparticles (NPs) and drug delivery systems for targeted therapies, including liposomes, polymeric nanoparticles, and nanodiamonds [122].

## 6. Discussion

HER2 was discovered in the 1980s [5,6]. Since then, HER2 has become a significant therapeutic target for breast (OE in around 30%) and gastric cancers (OE in around 20%) [123,124]. HER2 aberrations are also present in NSCLC and consist of three distinct mechanisms: gene mutation (1–4%), gene amplification (2–20%), and protein overexpression (2–35%) [2]. Although all of the HER2 alterations were demonstrated to be a potential negative prognostic factor in patients with NSCLC for survival both in early and advanced diseases, aiming to target these alterations in NSCLC had disappointing outcomes for decades [18,21,22].

HER2 mutations within the TKD were first discovered in NSCLC in 2004 [11]. They were described in 4.2% in NSCLC, but as enriched among lung adenocarcinoma (~10%) [11]. The oncogenic (or likely oncogenic) mutations in TKD are around 74%. These mutations occur mainly in Ex20 (90%), with the most frequent being A775_G776insYVMA, which represents almost half of the cases (43%), followed by Ex19 (~6%), Ex18 (~4%), and Ex21 (<1%) [49,50]. Beyond these mutations, there are also mutations in the ECD (21%), TMD (5%), and non-TKD intracellular domains (<1%) [34,52]. Ex20 insertions in the HER2 gene result in a conformational change in the TKD that led to an increase kinase activity compared to HER2 WT and confers resistance to the common EGFR/HER2 TKI [8,51]. Retrospective small-series studies depict the clinical features of patients with HER2 MUT NSCLC: younger (middle age around 60 years), female sex, adenocarcinoma histology, light or never-smokers, and generally mutually exclusive of other molecular drivers [12,13,14,15,16,17,18,19]. Very rarely, they can be associated with other concomitant mutations [17]. Thus, HER2 gene mutation has emerged as an oncogenic factor and therapeutic target in lung cancer, for which guidelines recommend testing in advanced NSCLC [25,125].

The first therapeutic attempts with targeted therapy derive from mAbs directed toward HER2 (trastuzumab and then pertuzumab), which, when administered alone or in combination with chemotherapy, have not demonstrated activity either in pretreated or naïve patients, nor in patients with HER2 OE or AMP NSCLC; however, above all, they have also been disappointing even in patients with HER2 MUT NSCLC [53,54,55,56,57,58,59,60,61].

A field of intense research is represented by TKIs. The development of selective HER2 inhibitors sparing EGFR WT inhibition was really hard because EGFR and HER2 shares the same sequence homology within the TKD [126]. Moreover, the tridimensional structures of the most frequent mutations within the TKD (Ex20 insertions) are similar to that of EGFR and HER2 WT structures; therefore, it was necessary to design molecules with high specificity towards HER2 MUT to improve both their efficacy and, above all, their safety profile, which are linked to the inhibition of EGFR WT [127]. From the beginning, the pan-HER TKIs were explored, and numerous studies were conducted with different drugs; however, given their non-specificity (against to EGFR/HER2/HER4), the results were only modest and were associated with a high incidence of EGFR-related toxic effects, such and diarrhoea and skin rash [62,63,64,65,66,67,68,70]. Then came the EGFR/HER2 TKIs, which showed a more interesting activity; however, even these remained limited by EGFR-related skin and gastrointestinal toxicity [72,73,74,75]. However, BAY2927088 has recently emerged as promising drug because it presents different characteristics (it is a potent non-covalent and reversible inhibitor with low activity on the EGFR WT protein), so this has led to a reduction in toxicity—at least cutaneous toxicity—although patients still experienced a G ≥ 3 rate around 50% [76,83]. Furthermore, impressive results derived from the new HER2-selective TKIs, such as zongertinib (ORR 71% ≥ 2 L), which, being highly specific for the HER2 mutation and with minimal effects on the EGFR WT receptor, have significantly reduced the grade of gastrointestinal and skin AEs with a G ≥ 3 around 15% [78,85]. Interestingly, both drugs (BAY2927088 and zogertinib) proved to be active toward patients with HER2 MUT NSCLC previously treated with anti-HER2 ADCs [78,83]. So, in 2024, BAY2927088 and zongertinib received the BTD from the FDA for pretreated patients with HER2 MUT NSCLC [82,86].

In the meantime, immunotherapy has been used in real-world scenarios for patients with HER2-alterated (MUT or AMP) NSCLC, in pretreated patients and then in first-line therapy associated with chemotherapy (data derive from retrospective studies) [101,102,103,104,105,106,107,108,109,110,111,112]. Although the response to single ICI therapy is lower in patients that were extensively pretreated, many studies have demonstrated a positive association with positive/high PD-L1, high TMB, and smoking status [102,103,104,105,106,107,108,110].

In 2022, the first drug targeted for HER2 (T-DXd) was approved in patients previously treated with HER2 MUT NSCLC [29]. Indeed, the SoC for treatment of naïve patients with NSCLC HER2 alterations (including also HER2 MUT) is still chemotherapy, associated with ICI producing non-lasting responses (up to 60% with a mPFS around 6 months) [26,27,28]. The FDA/EMA approval of T-DXd was based on the results from DESTINY-Lung 01 and -Lung 02 trials, where the drug attained, in pretreated patients and at the approved dosage (5.4 mg/kg every 3 weeks), an ORR of 50% with a mPFS 10.0 months and, importantly, halved the occurrence of ILD respect to the higher dose (6.4 mg/kg), 14% vs. 32%, respectively [95,98]. Interestingly, the drug also showed activity in pretreated patients with HER2 OE NSCLC (DESTINY-Lung01 cohort 1), with ORRs around 30% and mPFS around 6 months, but did not attain an approval for this subgroup of patients [96]. The current and future developments of T-DXd will include investigations of the activity of this drug toward brain metastases (phase II trial ELPIS, NCT06250777) or in the first-line setting administered alone in two phase III trials controlled for SoC chemotherapy + ICI in patients with HER2 MUT NSCLC (DESTINY-lung04) (NCT05048797), or with HER2 OE/PD-L1 intermediate NSCLC (DESTINY-Lung06) (NCT06899126), or in association with rilvegostomig with or whithout carboplatin (phase Ib DESTINY-Lung03 part 4) in patients with HER2 OE NSCLC [120].

Brain metastases remain a challenge for HER2-positive patients. The assessment of intracranial activity in anti-HER2 therapies is of paramount importance. Considering that many patients have up to 47% brain metastases, it is essential to achieve the optimal control of brain disease and to delay brain irradiation, taking into account the potential side effects of this treatment. In the DESTINY-Lung01, 33 patients with stable and asymptomatic brain metastases treated with T-DXd obtained an ORR of 54.5% and mPFS of 7.1 months [95]. Also, in the HORIZON-Lung study, 24 Chinese patients with stable brain metastasis treated with trastuzumab rezetecan obtained an ORR of 87.5% and mPFS of 9.9 months [99]. Instead, the Beamion-Lung 01 trial, which evaluated the IC-ORR according to RANO-BM criteria, reported an IC-ORR of 41% [84]. Also, currently ongoing is a phase II ELPIS trial that has, as its primary endpoint, IC-PFS, defined by RANO criteria, in patients with asymptomatic brain metastases treated with T-DXd. In this direction, many other studies with new selective HER2 TKI have, as a primary or secondary endpoint, efficacy in CNS (IC-ORR or IC-PFS) (HEROEX-1; IAM1363-01, NCT06253871; and ORIC-114, NCT05315700). Furthermore, preclinical studies are underway to overcome resistance to radiotherapy [128].

Regarding the new promising therapies, a wave of new drugs (new selective TKI, new ADCs, bsAbs, bsADC, vaccines, and others) directed against HER2 is blooming and, therefore, contribute to the great expectation of finding the best therapy for this group of patients, whose survival remains poor.

Therefore, in the near future, we will witness the results of three important phase III trials of T-DXd, BAY2927088, and zongertinib controlled with SoC (chemotherapy plus ICI) in the treatment of naïve HER2 MUT NSCLC patients. However, considering the complexity of this type of disease, together with the results already realised in the EGFR Ex20ins NSCLC disease, where the current standard is represented by the addition of amivantamab to the standard chemotherapy, we believe that future developments in the research of HER2 MUT NSCLC will be around the design of the following new combinations: TKI or ADC plus chemotherapy +/− ICI [129].

## 7. Conclusions

HER2 mutations can be considered effective oncogenic drivers in NSCLC; therefore, molecular testing for them is recommended in advanced disease. T-DXd has been approved by the FDA and EMA in pretreated patients with HER2-mutant NSCLC; however, it is not used in all European countries due to delays in the approval by local agencies. Given the encouraging results obtained in pretreated patients, we have great expectations for a first-line phase 3 study that compared T-DXd to SoC (chemoimmunotherapy). The two newly HER2-specific TKIs with low EGFR WT inhibition (BAY2927088 and zongertinib) have shown high efficacy with better tolerability and have currently obtained BTD from the FDA. A strong commitment to translational research and the development of new drugs and/or new combinations active against HER2-positive NSCLC will allow us to further improve these encouraging results and reach ‘the long way to the top’.

## Figures and Tables

**Table 1:** Summary of phase II trials with monoclonal antibodies alone or in combination with chemotherapy in patients with HER2-altered NSCLC.

Reference	Histology	Setting	Treatment	HER2 Testing	Pt N	ORR (%)	DCR (%)	mPFS (mos)	mOS (mos)
Kinoshita (2018) [53] [HOT1303.B]	ADK	>2 L	Trastuzumab	HER2 OE (3+/2+ DISH+) HER2 MUT	10 ^$^	0	70	5.2	-
Lara (2004) [54]	NSCLC	≥2 L	Trastuzumab + docetaxel	HER2 OE	13	8	-	4.3	5.7
Gatzemeier (2004) [55]	NSCLC	1 L	Trastuzumab + Cisplatin/gemcitabine	HER2 OE or HER2 AMP	51	36	80	6.1	-
			Cisplatin/gemcitabine		50	41	94	7.0	-
Zinner (2004) [56]	NSCLC	1 L	Trastuzumab + Cisplatin/gemcitabine	Not selected IHC (0–3+) ^£^	21 (9 *)	38	71	8.5	70 + weeks
Langer (2004) [57]	NSCLC	1 L	Trastuzumab + carboplatin/paclitaxel	Not selected IHC (1+/2+/3+)	53	24.5		3.3	10.1
Krug (2005) [58]	NSCLC	1 L	Trastuzumab + weekly docetaxel	Not selected IHC (0–3+)	30 (9 *)	23	63	-	16
			Trastuzumab + weekly paclitaxel		34 (11 *)	32	58	-	14
Van Berge HJM (2022) [59]	NSCLC	≥2 L	Trastuzumab + pertuzumab	HER2 MUT **	24	8.3	38	4	10
Hainsworth (2018) [60] [MyPathway study]	NSCLC ^&^	≥2 L	Trastuzumab + pertuzumab	HER2 OE (3+) AMP or GCN	16	13	46	-	-
				HER 2 MUT	14	21	42	-	-
Mazieres (2022) [61] IFCT-1703 R2D2 Trial	NSQ-NSCLC	≥1 L	Trastuzumab + pertuzumab + docetaxel	HER2 MUT (Ex20 ins)	45	29	87	6.8	17.6

Legend: HER2 OE-defined tumours with HER2 scores of 2 or 3 positivity, except as otherwhere indicated; ^$^ 3 pts were overexpressed and 7 pts were mutated (5 insertion exon20 and 2 310F); ^£^ included patients with 0 or not available IHC; * HER2-positive pts (IHC 2+ or 3+); ** 71% of patients had HER2 mutation p.Y772-A775dup; ^&^ Basked trial in all solid tumours; we only report the group lung cancer. Abbreviations: Pt N = patient number; ORR = overall response rate; mPFS = median progression-free survival; mOS = median overall survival; mos = months; ADK = adenocarcinoma; NSCLC = non-small cell lung cancer; NSQ = non squamous; DCR = disease control rate; HER2 = human epidermal growth factor receptor 2; OE = overexpressed; AMP = amplified; MUT = mutated; IHC = immunohistochemistry; DISH = dual colour in situ hybridization; Ex20 = exon 20; ins: insertion.

**Table 2:** Summary of trials with HER2-directed TKIs (pan-HER TKIs, EGFR/HER2 TKIs, and selective HER2 TKIs).

Ref.	Drug	Trial Phase	Histology	Setting	Pt N	Type of HER2	ORR (%)	DCR (%)	mDOR (mos)	mPFS (mos)	mOS (mos)	Safety (Most Common AEs)
**Pan-HER TKIs**												
Kris (2015) [62]	Dacomitinib	II	ADK	≥1 L ^$^	30	MUT (26) °, AMP (4)	12 ^#^	60 ^#^	-	3 ^#^	9 ^#^	Diarrhoea 90% (G ≥ 3 23%), skin rash 73% (G ≥ 3 3%).
Gandhi (2017) [63] PUMA-NER-4201	Neratinib	II	NSCLC	≥1 L	17	MUT (Ex20 95%)	0	35	0	2.9	10	Diarrhoea 82% (G ≥ 3 12%) ^@^, stomatitis 6% (G ≥ 3 0%).
	Neratinib + temsirolimus				43		8	49	2.4–22.6	4.0	15.8	Diarrhoea 86% (G ≥ 3 14%) ^@^, stomatitis 49% (G ≥ 3 7%).
Li (2021) [64] PUMA-NER-5201 (SUMMIT)	Neratinib	II (basket)	NSCLC	≥2 L	26	MUT (Ex20 66%)	4	39	9.2	5.4	-	Anaemia 13%, diarrhoea 66%, costipation 35%, nausea 41%, vomiting 33%.
	Neratinib + trastuzumab				52		8	27	4.0–18.3	4.1	-	Anaemia 18%, diarrhoea 80%, costipation 30%, nausea 46%, vomiting 45%.
Dziadziuszko(2019) [65] NICHE trial	Afatinib	II	NSCLC	≥2 L	13	MUT Ex20	7.7	54	-	3.7 (15.9 weeks)	13.1 (56 weeks)	Gastrointestinal AEs, skin rash, paronychia, mucositis.
Fan (2020) [66]	Afatinib	II (part A ^£^)	NSCLC		18	MUT Ex19–20	0	61	-	2.8	10.0	Diarrhoea 66.7% (G ≥ 3 16.7%), skin rash 33% (G ≥ 3 0%), transamitis 38.9% (G ≥ 3 5.6%).
Robichaux (2019) [49]	Poziotinib	II	NSCLC	≥2 L	12	MUT Ex20 ins	42	83	-	5.6	-	Diarrhoea 69% (G ≥ 3 17%), skin rash 42% (G ≥ 3 58%), nausea 31% (G ≥ 3 8%).
Le (2022) [67] ZENITH20–2	Poziotinib	II	NSCLC	≥2 L	90	MUT Ex20 ins	27.8	70.0	5.1	5.5	-	Skin rash 91.1% (G ≥ 3 48.9%), diarrhoea 82.2% (G ≥ 3 25.6%), stomatitis 68.9% (G ≥ 3 24.4%).
Cornelissen (2023) [68] ZENITH20–4	Poziotinib	II	NSCLC	1 L	80	MUT Ex20 ins	39	73	5.7	5.6	-	Skin rash 98% (G ≥ 3 45%), diarrhoea 83% (G ≥ 3 15%), stomatitis 81% (G ≥ 3 21%).
Elamin (2022) [69]	Poziotinib	II	NSCLC	≥2 L	30	MUT Ex20 ins	27	73	5.0	5.5	15	Skin rash 83% (G ≥ 3 47%), diarrhoea 80% (G ≥ 3 20%), stomatitis 67% (G ≥ 3 10%).
Liu (2020) [70] RAIN-701	Tarloxitinib	II (cohort B)	NSCLC	≥2 L	11	MUT	22	66	-	-	-	Prolonged QTc 60.9% (G≥ 3 34.8%), rash 43.5% (G ≥ 3 4.3%), diarrhoea 21.7% G ≥ 3 4.3%).
**EGFR/HER2 TKIs**												
Ross (2010) [71]	Lapatinib	II	NSCLC	1–2 L	56 ^ç^	MUT and AMP	0	-	21–28	8.7–15.6 weeks	55.4–60.9 weeks	Diarrhoea, rash, fatigue, nausea, anorexia.
Wang (2019) [72]	Pyrotinib	II	NSCLC	≥2 L	15	MUT Ex20	53	83	7.2	6.4	12.9	Anaemia 27%, hypocalcemia 27%, rash 14%, nausea 7%.
Song (2022) [73]	Pyrotinib	II	ADC	≥1 L	78	Mutations (Ex20 and not Ex20)	19.2	74.4	9.9	5.6	10.5	Diarrhoea 85.9% (G ≥ 3 16.7%), anaemia 35.9% (G ≥ 3 2.6%), fatigue 57.7% (G ≥ 3 1.3%).
Liu (2023) [74]	Pyrotinib	II	NSCLC	1 L	28 *	MUT	37.5	89.3	6.4	7.3	14.3	Diarrhoea 85.7% (G ≥3 0%), rash 32.1% (G ≥ 3 0%), creat. increased 16.7% (G≥ 3 0%).
					12 **		16.7	83.4	(4.6 + −10.5)	4.7	14.2	Diarrhoea 91.7% (G ≥ 3 16.7%), rash 4.7% (G ≥ 3 0%), increased AST 25% (G≥ 3 3.6%).
					8 ***		0	75.0	-	3.0	12.2	na
Kanemura (2024) [75]	Mobocertinib + TDM1	Ia/Ib	NSCLC	NS	13	MUT exon 20 ins YVMA	53.8	84.6	-	6.1	-	Thrombocytopenia 50.0%, diarrhoea 13.6%, anorexia, 13.6%, no ILD.
Le (2024) [76] SOHO-01	BAY2927088	I/II (cohort D)	NSCLC	≥ 2 L	44	MUT	72.1	83.7	8.7	7.5	-	Diarrhoea 86.4% (G ≥ 3 25%), rash 43% (G ≥ 3 0%), paronychia 25% (G ≥ 3 0%), no ILD.
**Selective HER2 TKIs**												
Reck (2021) [77] SGNTUC-019	Tucatinib	II	All solid tumours (NSCLC)	≥ 2 L	217	OE/AMP cohort and MUT cohort	NP	NP	NP	NP	NP	NP
Heymach (2025) [78] Beamion LUNG-1	Zongertinib ^@^	Ia/Ib Cohort 1	NSCLC	≥ 2 L	75	MUT TKD	71	95	14.1	12.4	-	Diarrhoea 56% (G ≥ 3 1%), rash 33% (G≥3 0%), ALT increased 21% (G ≥ 3 8%).
		Ia/Ib Cohort 3	NSCLC	≥ 2 L	20	MUT non-TKD	30	65	NE °°	NE °°	-	Diarrhoea 50% (G ≥ 3 5%), rash 15% (G ≥ 3 0%), nausea 25% (G ≥ 3 0%).
		Ia/Ib Cohort 5	NSCLC	≥ 2 L (treated with ADC)	22 ^&^ (31)	MUT TKD	41	-	5.3	6.8	-	Diarrhoea 45% (G ≥ 3 0%), rash 19% (G ≥ 3 0%), nausea 26% (G ≥ 3 3%).

Legend: ^ç^ all HER2- and EGFR-positive NSCLC; ^@^ used loperamide prophylaxis; ^$^ 17% first-line treatment and 83% beyond; * cohort of criteria fulfilled (CF); ** cohort of compassionate use (CU); *** cohort of real-world-studies (RWS); ° all patients have HER2 mutations in exon 20 (25 insertion); ^#^ results of HER2-mutated populations (n = 26); ^£^ Part B was not performed with afatinib + paclitaxel for no responses in Part A; ^@^ at a dose of 120 mg once daily; ^&^ patients treated with trastuzumab deruxtecan; °° not evaluable because not yet mature. Abbreviations: Pt N = patient number; ORR = overall response rate; DCR = disease control rate; DOR = duration of response; mPFS = median progression-free survival; mOS = median overall survival; mos = months; ADK = adenocarcinoma; NSCLC = non-small cell lung cancer; HER2 = human epidermal growth factor receptor 2; OE = overexpressed; AMP = amplified; MUT = mutated; Ex = exon; ins: insertion; TKD: tyrosine kinase domain; NE: not estimable; NP: not published; na = not available.

**Table 3:** Summary of trials with ADC in patients with HER2 altered NSCLC.

Ref.	Drug	Trial Phase	Histology	Setting	Pt N	HER2 Alteration	ORR (%)	mDOR (mos)	mPFS (mos)	mOS (mos)	FUP	Safety (Most Common AEs)
Li (2018) [90]	TDM1	II	ADK	≥2 L	49	28 MUT, 11 AMP, 10 both	51	4.4	5.0	NR	NR	Transaminitis 63% (G ≥ 3 0%), thrombocytopenia 31% (G ≥ 3 2%), fatigue 16% (G ≥ 3 0%), nausea 29% (G ≥ 3 0%).
						28 MUT	50	4.0	5.0	NR	NR	Transaminitis 44% (G ≥ 3 0%), thrombocytopenia 33% (G ≥ 3 0%), fatigue 33% (G ≥ 3 0%), nausea 33% (G ≥ 3 0%).
Hotta (2018) [91]	TDM1	II	NSCLC	≥2 L	15 *	5 pts IHC 3+, 3 pts IHC 2+/FISH+	0	NR	2.0 ^$^	10.9 ^$^	9.2 ^$^	G ≥ 3: thrombocytopenia 40%, AST/ALT increases 7%, hyperuricemia 7%, nausea 7%.
						7 pts MUT (Ex20)	14.3	NR				
Peters (2019) [92]	TDM1	II	NSCLC	≥2 L	49	29 IHC 2+	0	0	2.6	12.2	23.1	Any AEs: 45% (G ≥ 3 11%): haemorrhage 7% (G ≥ 3 0%), peripheral neuropathy 7% (G ≥ 3 0%), thrombocytopenia 4% (G ≥ 3: 1%).
						20 IHC 3+	20	2.9–10.8	2.7	15.3	18.4	
Iwama (2022) [93]	TDM1	II	ADK	≥2 L	22	MUT (Ex20)	38.1	3.5	2.8	8.1	8.0	Thrombocytopenia 63.6% (G ≥ 3 18.2%), transaminitis 43% (G ≥ 3 0%), nausea 31% (G ≥ 3 0%).
Tsurutani (2020) [94]	T-DXd	I	NSCLC ^&^	≥2 L	18	MUT 11 pts; OE 18 pts (16 pts IHC 3+ and 2 pts IHC 2+).	55.6	10.3	11.3	NE (17.3-NE)	11	G ≥ 3: anaemia 25.4%, neutropenia 20.3%, leucopenia 18.6, thombocytopenia 15.3%, febbrile neutropenia 5.1%. ILD G1 (1.7%).
Li (2022) DESTINY-Lung01 [95]	T-DXd	II (cohort 2)	NSCLC	≥2 L	91 (6.4 mg/kg)	MUT (OE/AMP) °	55	9.3	8.2	17.8	13.1	Nausea 73% (G ≥ 3 9%), fatigue 53% (G ≥ 3 7%), vomitinig 40% (G ≥ 3 0%), neutropenia 35% (G≥3 19%), ILD 26% (G1-2: 20%, G3: 4%, G5: 2%).
Smit (2024) [96] DESTINY-Lung01	T-DXd	II (cohort 1)	NSCLC	≥2 L	49 (6.4 mg/kg)	OE (IHC 2+/3+)	26.5	5.8	5.7	12.4	12.0	G ≥ 3: 53%. Nausea 59% (G ≥ 3 6%), fatigue 59% (G ≥ 3 12%), vomiting 31% (G ≥ 3 4%), neutropenia 25% (G ≥ 3 24%), ILD 20% (G ≥ 3 6%).
		II (cohort 1A)			41 (5.4 mg/Kg)	OE (IHC 2+/3+)	34.1	6.2	6.7	11.2	10.6	G ≥ 3: 22%. Nausea 73% (G ≥ 3 5%), fatigue 70% (G ≥ 3 7%), vomiting 29% (G ≥ 3 2%), neutropenia 10% (G ≥ 3 0%), ILD 5% (G ≥ 3 2%).
Goto (2023), [97] Janne (2024) [98] DESTINY-Lung02	T-DXd	II	NSCLC	≥2 L	102 (5.4 mg/Kg)	MUT	50	12.6	10.0	19.0	15.8	G ≥ 3: 39%. Nausea 67.3% (G ≥ 3 4%), neutropenia 42.6% (G ≥ 3 18.8%), anaemia 36.6% (G ≥ 3 10.9%), fatigue 44.6% (G ≥ 3 7.9%), ILD 14% (G ≥ 3 2%).
					50 (6.4 mg/kg)	MUT	56	12.2	12.9	17.3	16.5	G≥3: 60%. Nausea 82% (G ≥ 3 6%), neutropenia 56% (G ≥ 3 36%), anaemia 52% (G ≥ 3 16%), fatigue 50% (G ≥ 3 10%), ILD 32% (G ≥ 3 2%).
Li (2025) [99] HORIZON-Lung	SHR-A1811	II	NSCLC	≥2 L	94 (4.8 mg/Kg)	MUT (IHC 0, 1+, 2+) °	73	NE (8.3-NE)	11.5	NE ^§^	8.7	G3-4: neutropenia 40%, leucopenia 27%, anaemia 23%, thrombocytopenia 11%, ILD 7%.

Legend: HER2 OE-defined tumours with HER2 scores of 2 or 3 positivity, except as otherwhere indicated; * this study was terminated early because of the limited efficacy of treatment; ^$^ result in all OE and MUT patients (n = 15), ^&^ of all solid tumours, we report only the patients with non-small cell lung cancer (NSCLC); ° OE or AMP was evaluated in exploratory analysis, and is not required for the inclusion criteria; ^§^ not mature. Abbreviations: Pt N = patient number; ORR = overall response rate; mDOR = median duration of response; mPFS = median progression-free survival; mOS = median overall survival; mos = months; FUP = median follow-up; AEs = adverse events; G = grade; ADK = adenocarcinoma; NSCLC = non-small cell lung cancer; HER2 = human epidermal growth factor receptor 2; OE = overexpressed; AMP = amplified; MUT = mutated; IHC = immunohistochemistry; FISH = fluorescence in situ hybridization; Ex20 = exon 20; NE: not estimable; pts = patients; NR = not reported; ILD = interstitial lung disease; L = line of treatment.

**Table 4:** Summary of studies including immunotherapy in patients with HER2-altered NSCLC.

Ref.	Country	Study	Time	Line	HER2 Alteration	Pt N	ORR (%)	mPFS (95% CI) mos	mOS (95% CI) mos	Predictive Factors
Dudnik 2018 [101]	Israel	R	2013–2017	1–4 L	AMP	5	12	6.3 (1.8–9.3)	10.4 (2.2-NR)	No correlations with PD-L1, TMB, or smoking.
					MUT	9	20	3.4 (2.4–8.5)	17.5 (3.0–17.5)	
Lai 2018 [102]	USA	R	NA	NA	MUT (Ex 7, 8, 16, 17, 19, 20)	26	16	1.9 (1.5–4.0)	10.4 (5.9-NR)	Correlation with PD-L1 and high and median TMB.
Negrao 2018 [103] (MDACC)	USA	R	NA	≥1 L	MUT (Ex20)	16	6	1.8	17.1	Correlation with high PD-L1.
Mazieres 2019 [104] [IMMUNOTARGET]	Europe	R	2017–2018	≥1 L	MUT (Ex20)	29	7.4	2.5 (1.8–3.5)	21.3 (3.2–18.0)	Smoking status/PD-L1 positive (>1%).
Guisier 2020 [105]	France	R	NR	2–4 L	MUT (Ex20)	23	27.3	2.2 (1.7–15.2)	20.4 (9.3-NR)	Correlation with positive PD-L1 and early treatment line.
Lau 2021 [106]	Canada	R	2013–2019	2 (1–4) L	MUT: Ex20 (8 pts), Ex 17–19 (6 pts)	14	29	3.6 (1.6-NR)	5.9 * (3.4–12)	PD-L1 as an independent predictor of improved PFS.
Negrao 2021 [107]	USA	R (MDACC)	2014–2018	≥1 L	MUT (codons 755 and 770–785)	15	8	1.88	16.8	Positive association between PFS and OS with positive PD-L1.
	USA	R (CGBD)	2011–2018	≥1 L	MUT (codons 755 and 770–785)	28	NA	3.02	10.81	Positive association between PFS and OS with postive PD-L1 and TMB ≥10 mt/Mb.
Saalfeld 2021 [108]	Germany	R	2016–2020	1 L (CT + IO)	MUT (Ex 20, 19, 8)	22	52	6 (6–14)	NE (immature)	Correlation with PD-L1 in first-line therapy with CT-IO.
				1 L (IO)		5	25	NA	NA	-
				≥ 2 L (IO)		34	16	4 (4–6)	10 (6-NA)	-
DeMatteo (2022) [109]	USA	R	2014–2021	≥ 1 L	AMP (NGS)	18	0	2 (1–7)	11 (4–37)	No correlations with high PD-L1 (n = 3) and TMB ≥ 10 mt/Mb (n = 9).
Tian 2021 [110]	China	R	2015–2019	1–2 L (CT + IO)	MUT (Ex20 ins)	13	31	8.0 (5.2-NR)	NA	Correlation with high TMB, DNA MMR, and SWI/SNF complex.
Yang 2022 [111] [POLISH]	China	R	2015–2021	1 L (CT + IO)	MUT (42 pts), AMP (5 pts)	46	28.9	5.2 (3.64–6.76)	NE (immature)	No correlations with high PD-L1 and high TMB.
				1 L (CT + A)	MUT (78 pts), AMP (3 pts)	81	23.8	5.63 (4.84–6.43)	36.27 (28.71–42.83)	
				1 L (CT)	MUT (78 pts), AMP (5 pts)	83	16.9	4.03 (2.70–5.37)	31.67 (29.63–33.71)	
Chu 2022 [112]	China	R	2016–2021	≥1 L (CT + IO)	MUT	16	60	8.4	NA	Lower proportion of female patients (6/16).
				≥2 L (IO)		5	40	5.3	NA	No association with PD-L1 or smoking status.

Legend: * all the population. Abbreviation: R: retrospective, NA: not available, NR: not reached; NE: not estimable; Mut: mutations; Mb: megabasis; Pt N = patient number; ORR = overall response rate; mPFS = median progression-free survival; mOS = median overall survival; mos = months; NSCLC = non-small cell lung cancer; HER2 = human epidermal growth factor receptor 2; AMP = amplified; MUT = mutated; Ex = exon; ins: insertion; NGS = next generation sequencing; L = line of treatment; PD-L1 = Programmed Death-Ligand 1; TMB= tumour mutational burden; CT= chemotherapy; IO = immunotherapy; DNA= deoxyribonucleic acid; MMR = DNA mismatch repair, SWI/SNF = mammalian SWItch/Sucrose Nonfermentable chromatin remodelling complexes.

## Data Availability

No new data were created in this study. Data sharing is not applicable to this article.

## Supplementary Materials

The following supporting information can be downloaded at: https://www.mdpi.com/article/10.3390/molecules30122645/s1. Table S1. Clinical features of patients with HER2 mutant lung adenocarcinoma; Table S2. HER2 mutations (oncogenic and likely oncogenic) in 35 studies of NSCLC of cBioportal; Table S3. Characteristics of drugs of the three class of HER2 TKIs (pan-HER TKI, EGFR/HER2 TKI and selective HER2 TKI); Table S4. Features of ADCs investigated in HER2-alterated NSCLC; Table S5. Summary of ongoing clinical trials of HER2 positive NSCLC.
