# Supplementary material for: HER2 in Non-Small Cell Lung Cancer (NSCLC): Evolution of the Therapeutic Landscape and Emerging Drugs—A Long Way to the Top
Source: Molecules. 2025 Jun 18;30(12):2645. doi: 10.3390/molecules30122645 (PMC12195848; doi:10.3390/molecules30122645)
Supplement: Supplementary file 1 [file molecules-30-02645-s001.zip › molecules-3656752-supplementary.pdf]

HER2 in Non-Small Cell Lung Cancer (NSCLC): Evolution of the Therapeutic Landscape and Emerging Drugs—A Long Way to the Top

Pamela Trillo Aliaga[1,*], Gianluca Spitaleri[1], Ilaria Attili[1], Carla Corvaja[1], Elena Battaiotto[2,3], Panagiotis Agisilaos Angelopoulos[2,3], Ester del Signore[1], Antonio Passaro[1,*], and Filippo de Marinis[1].

Table S1: Clinical features of patients with HER2 mutated lung adenocarcinoma

Reference	Country	Pt N	Mutation	Median Age (years)	Female sex (%)	Never smoker (%)	Notes
Shigematsu 2005	Japan/Taiwan/ Australia	11	Ex 20 (dup/ins)	64.5	63.5%	82%	-
Buttita 2006	Italy	9	7 pts Ex 20 (dup/ins)<br>1 pts Ex 20 (sub)<br>1 pts Ex 19 (sub)	NR	44.5%	33.3%	Negative for EGFR/KRAS
Arcila 2012	USA	25	24 Exon 20 (ins)	64	68%	68%	Negative for EGFR/KRAS/ALK<br>HER2 AMP 0%
Li 2012	China	8	Ex 20 (ins)	52	87.5%	100%	High HER2 GCN 87.5%<br>HER2 AMP 50%
Mazieres 2013	France/ Switzerland/Spain	65	Ex 20 (ins)	60	69%	52.3%	HER2 AMP 9%
Song 2016	China	21	Ex 20 (ins)	60	66.5%	81%	2 pts with SCC<br>HER2 AMP 4.5%<br>Concomitant mut
Kim 2017	Korea	7	6 pts Ex20 (ins)<br>1 pts Ex20 (sub-ins)	61	85.5%	85.5%	HER2 AMP 57%
Bu 2017	China	35	Ex 20 (ins)	60	91.4%	97.1%	2 pts with SCC

Abbreviations: Pt N = patient number; EX = exon; dup = duplication; ins = insertion; sub= substitution; EGFR = human epidermal growth factor receptor; HER2 = human epidermal growth factor receptor 2; HER2 AMP = HER2 amplification; GCN = gene copy number; SCC = squamous cell carcinoma; mut = mutations; USA: United states of America; pts =patients; KRAS =Kirsten rat sarcoma virus; ALK = Anaplastic Lymphoma Kinase.

Table S2: HER2 mutations (oncogenic and likely oncogenic) in 35 studies of NSCLC of cBioportal

Region	Exon	Protein MUT	cbioportal (n.cases)	N (%)	Annotation OncoKB	Mutation Type	Sensitivity in vitro	Sensitivity in patients
ECD	1	R103Q	14	4,1	Likely Oncogenic, level_1	Missense_Mutation	na	na
ECD	2	G222C	1	0,3	Likely Oncogenic, level_1	Missense_Mutation	na	na
ECD	none	V272G	1	0,3	Likely Oncogenic, level_1	Missense_Mutation	na	na
ECD	2	D277Y	11	3,2	Oncogenic, level_1	Missense_Mutation	neratinib (S), afatinib (S), trastuzumab (S)	na
ECD	6	A293P	1	0,3	Likely Oncogenic, level_1	Missense_Mutation	na	na

ECD	8	S310F	30	8,8	Oncogenic, level_1	Missense_Mutation	neratinib (S), afatinib (S), lapatinib (S), trastuzumab (S)	neratinib (S), T+P (S)
ECD	8	S310Y	3	0,9	Oncogenic, level_1	Missense_Mutation	neratinib (S), afatinib (S), lapatinib (S), trastuzumab (S)	na
ECD	none	A440P	1	0,3	Likely Oncogenic, level_1	Missense_Mutation	na	na
ECD	16	X633_splice	9	2,6	Likely Oncogenic, level_1	Splice_Site	afatinib (R)	
TMD	16	S649C	2	0,6	Likely Oncogenic, level_1	Missense_Mutation	na	na
TMD	16	S649T	1	0,3	Likely Oncogenic, level_1	Missense_Mutation	na	na
TMD	17	L651V	2	0,6	Likely Oncogenic, level_1	Missense_Mutation	na	na
TMD	17	V659D	1	0,3	Likely Oncogenic, level_1	Missense_Mutation	neratinib (S), afatinib (S), lapatinib (S), trastuzumab (S)	afatinib+apatinib (S)
TMD	17	V659E	6	1,8	Oncogenic, level_1	Missense_Mutation	trastuzumab (S)	neratinib (S)
TMD	17	G660D	1	0,3	Likely Oncogenic, level_1	Missense_Mutation	neratinib (S), afatinib (S), lapatinib (S), trastuzumab (S)	afatinib (S)
TMD (JMD)	none	E693K	1	0,3	Likely Oncogenic, level_1	Missense_Mutation	na	na
TMD (JMD)	20	Q709L	1	0,3	Oncogenic, level_1	Missense_Mutation	neratinib (S), afatinib (S), lapatinib (S), trastuzumab (S), pertuzumab (S)	
TKD	19	L755P	22	6,8	Oncogenic, level_1	Missense_Mutation	neratinib (R), afatinib (R), lapatinib (R)	neratinib (SD)
TKD	19	L755S	1	0,3	Oncogenic, level_1	Missense_Mutation	lapatinib (R), trastuzumab (R)	neratinib (S)
TKD	20	L755A	1	0,3	Likely Oncogenic, level_1	Missense_Mutation	poziotinib (S), tarloxitinib (R)	neratinib (PD)
TKD	20	L755F	2	0,6	Likely Oncogenic, level_1	Missense_Mutation	na	na
TKD	20	L755_N758delinsAKA	2	0,6	Oncogenic, level_1	In_Frame_Del	neratinib (S)	na
TKD	19	D769Y	2	0,6	Oncogenic, level_1	Missense_Mutation	neratinib (S), afatinib (S), lapatinib (S), trastuzumab (R)	neratinib (PD)
TKD	19	I767M	1	0,3	Likely Oncogenic, level_1	Missense_Mutation	neratinib (S), afatinib (S), lapatinib (S), trastuzumab (S)	na
TKD	20	E770_A771insGIRD	12	3,5	Oncogenic, level_1	In_Frame_Ins	neratinib (S), afatinib (S), lapatinib (R), trastuzumab (R)	na
TKD	20	A771_Y772insLRDG	6	1,8	Oncogenic, level_1	In_Frame_Ins	neratinib (S), lapatinib (S), trastuzumab (S)	neratinib (S),afatinib (S), lapatinib (S), trastuzumab (S), poziotonib (S), dacomitinib (S)
TKD	20	Y772_A775dup	146	42,9	Oncogenic, level_1	In_Frame_Ins	neratinib (S), afatinib (S), lapatinib (S), trastuzumab (S)	neratinib (S)
TKD	20	G776delinsAVGC	1	0,3	Oncogenic, level_1	In_Frame_Ins	neratinib (S), afatinib (S), trastuzumab (S)	na

TKD	20	G776S	1	0,3	Likely Oncogenic, level_1	Missense_Mutation	neratinib (S), afatinib (S), lapatinib (S), trastuzumab (S)	na
TKD	20	G776D	2	0,6	Likely Oncogenic, level_1	Missense_Mutation	neratinib (S) ,afatinib (S)	neratinib (S)
TKD	20	G776delinsVC	30	8,8	Oncogenic, level_1	In_Frame_Ins	neratinib (S),afatinib (S), lapatinib (R), trastuzumab (R)	neratinib (SD)
TKD	20	V777_G778insE	1	0,3	Oncogenic, level_1	In_Frame_Ins	neratinib (S), lapatinib (S), trastuzumab (S)	neratinib (S),afatinib (S), lapatinib (S), trastuzumab (S), poziotonib (S), dacomitinib (S)
TKD	20	V777L	2	0,6	Oncogenic, level_1	Missense_Mutation	neratinib (S), afatinib (S), lapatinib (S), trastuzumab (R)	neratinib (S)
TKD	20	V777M	1	0,3	Likely Oncogenic, level_1	Missense_Mutation	neratinib (S), afatinib (S), lapatinib (S), trastuzumab (S)	na
TKD	20	G778_P780dup	21	6,2	Oncogenic, level_1	In_Frame_Ins	neratinib (S), afatinib (R), trastuzumab (R)	neratinib (S)
total	-	-	340	100	-	-	-	-

Abbreviations: MUT= mutation; ECD= extracellular domain, TMD= transmembrane domain, JMD= juxtamembrane domain TKD =tyrosine kinase domain, S =sensitive, R =resistant, T =trastuzumab, P= pertuzumab, SD =stable disease, PD= progression of disease, na: not available.

Source:https://www.cbioportal.org/results/mutations?cancer_study_list=asclc_msk_2024%2Clung_msk_mind_2020%2Clung_smc_2016%2Clung_pdx_msk_2021%2Clung_msk_pdx%2Cluad_broad%2Cluad_cptac_2020%2Cluad_cptac_gdc%2Cluad_mskimpact_2021%2Cluad_mskcc_2020%2Cluad_msk_npjpo_2021%2Cluad_mskcc_2015%2Cluad_oncosg_2020%2Cluad_tcga%2Cluad_tcga_gdc%2Cluad_tcga_pub%2Cluad_tcga_pan_can_atlas_2018%2Cluad_tsp%2Cluad_mskcc_2023_met_organotropism%2Clung_nci_2022%2Clusc_cptac_2021%2Clusc_cptac_gdc%2Clusc_tcga%2Clusc_tcga_gdc%2Clusc_tcga_pub%2Clusc_tcga_pan_can_atlas_2018%2Cnsclc_ctdx_msk_2022%2Clung_msk_2017%2Cnsclc_mskcc_2018%2Cnsclc_pd1_msk_2018%2Cnsclc_mskcc_2015%2Cnsclc_tracerx_2017%2Cnsclc_unito_2016%2Cbm_nsclc_mskcc_2023%2Cnsclc_tcga_broad_2016&Z_SCORE_THRESHOLD=2.0&RPPA_SCORE_THRESHOLD=2.0&profileFilter=mutations%2Cstructural_variants%2Cna%2Cgistic&case_set_id=all&gene_list=ERBB2&geneset_list=%20&tab_index=tab_visualize&Action=Submit. Last access: 30 April 2025.

Table S3: Characteristics of drugs of the three class of HER2 TKIs (pan-HER TKI, EGFR/HER2 TKI and selective HER2 TKI)

**Drug**	**TKI class**	**Type of chemical bond**	**Type of inhibition**		
			**HER2 mut**	**HER2 wt**	**EGFR wt**
Dacomitinib	pan-HER	covalent irreversible	potent	potent	potent
Neratinib	pan-HER	covalent irreversible	potent	potent	potent
Afatinib	pan-HER	covalent irreversible	moderate	moderate	moderate
Poziotinib	pan-HER	covalent irreversible	potent	potent	potent
Tarloxotinib	pan-HER	covalent irreversible	potent	moderate	moderate
Lapatinib	EGFR/HER2	non-covalent reversible	potent	moderate	moderate
Pyrotinib	EGFR/HER2	covalent irreversible	potent	potent	potent
Mobocertinib	EGFR/HER2	covalent irreversible	potent	moderate	moderate
BAY2927088	EGFR/HER2	non-covalent	potent	-	minimal
Tucatinib	selective HER2	non-covalent reversible	potent	minimal	minimal
Zongertinib	selective HER2	covalent irreversible	potent	potent	minimal

Abbreviations: TKI: tyrosine kinase inhibitor; EGFR: epidermal growth factor receptor; HER2: Human epidermal growth factor receptor 2; mut: mutated; wt: wild type.

Table S4: Features of ADCs investigated in HER2-alterated NSCLC

Drug	class ADC	Payload	Type of payload	linker	DAR	Bystander effect
T-DM1	II	entamsine (DM1)	Antimicrotubule agent	non-clavable (thioether)	3.5	no
T-DXd	III	deruxtecan	topoisomerase I inhibitor	cleavable (tetrapeptide)	7.8	yes
SHR-A1811	III	rezetecan	topoisomerase I inhibitor	cleavable (tetrapeptide)	6.0	yes

Abbreviations: ADC = antibody drug conjugate; NSCLC = non-small cell lung cancer; T-DM: trastuzumab entamsine; T-DXd: trastuzumab deruxtecan; DAR: drug-to-antibody ratio (corresponding to the median number of payload molecules linked to each monoclonal antibody).

Table S5: Summary of ongoing clinical trials of HER2 positive NSCLC

Trial	phase	Drug Class	Target	Drug	Combination treatment or comparator arm	Line	Pts (estimated)	Primary endpoint	Secondary endpoints	Status
TKI										
CT06452277 (SOHO-02)	III	dTKI	HER2 MUT in TKD	BAY 2927088	versus SOC	1L	NSCLC, n= 278	PFS by BICR	PFS inv, ORR, DCR, DOR, OS, TAEs, QoL	R
NCT06521554 (HEROEX-1)	Ia/IIb	sTKI	HER2 (Ex20)	NVL-330	no	pre-treated	NSCLC, n=120	Safety, RP2D, MDT, TEAEs	ORR, DOR, IC-ORR, IC-DOR, TTR	R
NCT05650879	Ia/Ib	sTKI	HER2 MUT	ELVN-002	No (Part 1-3)	pre-treated	all tumours (NSCLC), n= 198	Safety (DLT, TEAEs)	ORR, DOR, PK parameters	R
		sTKI+ADC	HER2 MUT	ELVN-002	ELVN-002 +TDXd (Part4)					
NCT06253871	I/Ib	sTKI (irreversible)	HER2 alteration	IAM1363-01	no	pre-treated	all tumours (NSCLC), n=243	Safety (DLT, TEAEs), PK, ORR, IC-ORR	DOR, DCR, PFS, OS	R
NCT05435274	I/II	dTKI	EGFR/HER2 (Ex20)	HS-10376	no	pre-treated	NSCLC, n=380	MDT, ORR	TEAEs, DCR, DoR, PFS, OS, PK	R
NCT06706076 (SOLARA)	I/II	dTKI	EGFR/HER2 (exon 18-21)	BH-30643	no	pre-treated	NSCLC, n=266	Safety (DLT, RP2D) ORR	DCR, DOR, PFS, OS, QoL	R
NCT05532696	Ib/II	dTKI	HER2 (Ex20 ins)	ABT-101	no	pre-treated	all tumours (phase II: NSCLC), n=61	Safety (DLT, MDT, RP2D) ORR	DOR, DCR, PFS, OS	R
NCT06616766	I/II	dTKI	EGFR/HER2 MUT Ex20	YH42946	no	pre-treated	all tumors (NSCLC), n=161	Safety (TEAEs), ORR	PK, DoR, OS	R
NCT05315700	I/II	dTKI	HER2 MUT	ORIC-114	Alone (Part 1-11)	pre-treated	all tumors (NSCLC), n=350	RP2D, PK	ORR, DoR, CBR, PFS, icORR, icPFS	R
		dTKI	HER2 MUT	ORIC-114	ORIC-114+ CT (carboplatin/ pemetrexed) (Part III)					
NCT04982926	I	dTKI	EGFR/HER2 (exon 18-21)	TAS2940	no	pre-treated	all tumors (cohort A:NSCLC), n=29	MDT, ORR	PK, safety, DoR, DCR, PFS,	A,NR

NCT05364073	I	dTKI	HER2 MUT (Ex20 ins)	Furmonertinib (EGFR/HER2)	no	pre-treated	NSCLC (stage 2 - cohort2: HER2 MUT), n=170	Safety, PK, ORR	DOR, DCR, CNS-ORR, PFS, OS	A,NR
NCT03974022 (WU-KONG1)	I/II	dTKI	EGFR/HER2 MUT	DZD9008 (Sunvozertinib)	no	pre-treated (cohort 5 of Part A: naive)	NSCLC, n=315	Safety (part A), ORR (partB)	ORR, BOR, DoR, DCR,	A,NR
NCT05926180 (WU-KONG19)	I	dTKI	EGFR/HER2 MUT	DZD9008 (Sunvozertinib)	sunvozertinib + midazolam or digoxin or rosuvastatin	pre-treated	NSCLC, n=25	AUC, Cmax	NA	C
NCT03410927	I	dTKI	HER2/HER3 alterations	TAS0728	no	pre-treated	all tumours (group 5: NSCLC), n=19	Safety	PK, DCR, DoR, PFs, OS	T**
NCT04209465 (Master-Key01)	I	TKI	EGFR/HER/HER3 alterations	BDTX-189	no	pre-treated	all tumours (NSCLC), n=91	DLT, RP2D	TEAEs, PK, ORR, PFS	T[&]
combinations with TKI										
NCT06360211	I	dTKI	HER2 MUT	BAY2927088	BAY2927088 + midazolam	none	healthy, n=15	AUC, Cmax	TEAEs and safety	C
NCT06329895	I	dTKI	HER2 MUT	BAY2927089	BAY2927088 + dabigatran or rosuvastatine	none	healthy, n=15	AUC, Cmax	TEAEs and safety	C
NCT06348888	I	dTKI	HER2 MUT	BAY2927090	BAY2927088 + itraconazole or carbamazepine	none	healthy, n=30	AUC, Cmax	TEAEs and safety	C
NCT06378658	I	dTKI	HER2 MUT	BAY2927091	BAY2927088 + esomeprazole	none	healthy, n=21	AUC, Cmax	TEAEs	C
NCT04579380 (SGNTUC-019)	I	sTKI + mAB	HER2 alterations	tucatinib + trastuzumab	tucatinib +/- trastuzumab	pre-treated	all tumours (NSCLC), n=217	ORR	DCR, DoR, PFS, OS, safety	A, NR
NCT02834936	II	dTKI (pyrotinib)	HER MUT	pyrotinib	no	pre-treated	NSCLC, n=55	ORR	PFS	U
NCT05751018	II	dTKI (pyrotinib)	HER2 MU or AMP	pyrotinib	no	1L	NSCLC, n= 45	ORR	PFS, DCR, OS, AEs and SAEs	R
NCT04706949	II	dTKI + CT	HER2 MUT or AMP	pyrotinib	pyrotinib +carboplatin+ pemetrexed	1L	NSCLC, n=26	PFS	NA	U
NCT05016544	I/II	dTKI+ mAB	HER2 MUT or AMP	pyrotinib	pyrotinib + Inetetamab	pre-treated	NSCLC, n=48	DLT, SAEs	ORR, DCR, PFS, OS	U
NCT04382300	II	dTKI + IMA	HER2 MUT exon ins	pyrotinib	pyrotinib + thalidomide	pre-treated	NSCLC, n=39	ORR	PFS, OS, DCR, AEs, QoL	U
NCT04144569 (POETHIS)	II	dTKI + ICI	HER2 insertion	pyrotinib	pyrotinib + anti-PD1	pre-treated	NSCLC, n=30	PFS	ORR, OS	R
NCT02716116	I/II	dTKI	EGFR/HER2	TAK-788 (mobocertinib)	no	pre-treated	NSCLC, n=334	RP2D, ORR, icORR	Safety, PK, PFS, OS	A, NR
ADC										
NCT04818333	I/II	ADC	HER2 MUT, AMP, OE	SHR-A1811 (trastuzumab rezutecan)	no	pre-treated	NSCLC, n=157	Safety, MTD, RP2D, ORR	PK, PFS, DOR, DCR OS	A, NR
NCT06114511	Ib/II	ADC	HER2 MUT	BL-M17D1	no	pre-treated	NSCLC, n=58	RP2D (part Ib), ORR (part II)	TEAEs, DCR, DoR, PFS	R
NCT06496490	II	ADC	HER2 altered	TQB2102	no	pre-treated	NSCLC, n=270	ORR	DoR, PFS, OS, Safety, ADA	R

NCT05141786	II	ADC	HER2 (NGS/PCR)	MRG002	no	pre-treated	NSCLC, n=100	ORR by BICR	DCR, DOR, PFS, OS, safety	U
NCT04311034	I/II	ADC	HER2 (OE 2+ or 3+, MUT Ex20)	RC 48 (Disitamab Vedotin)	no	pre-treated	NSCLC, n=37	safety	ORR, DCR, PFS, DOR, OS	C
NCT06003231	II	ADC	HER2 (OE, MUT)	RC 48 (Disitamab Vedotin)	no	pre-treated	all tumours (cohort2: NSCLC), n=119	ORR by inv	AEs, DOR, DCR, PFS, OS	NR
NCT05514717	I	ADC	HER2 OE (2+ or 3+), AMP, MUT	XMT-2056	no	pre-treated	all tumours (cohort3: NSCLC) n=162	Safety (DLT, MTD, RP2D)	ORR, DOR, DCR	R
NCT04450732	I	ADC	HER2 OE, AMP, MUT	GQ1001	no	pre-treated	all tumours (NSCLC) n=96	DLT, MTD	PK, ORR, DOR, DCR, PFS	R
NCT06714617	I	ADC	HER2 expression (1+ to 3+) and MUT	BL-M17D1	no	pre-treated	all tumours (cohort3: NSCLC) n=120	DLT, AESs TEAEs	NA	NR
NCT05150691	I/IIa	ADC	HER2 alterated (OE, AMP, MUT)	DB-1303/BNT323	no	pre-treated	all tumours (NSCLC), n=796	Safety (DLT, MTD, RP2D) ORR	DCR, DOR, PFS, OS	R
NCT03125200	I	ADC	HER2	ADCT-502 (trastuzumab tesirine)	no	pretreated	all tumours (NSCLC) n=21	Safety	ORR, DCR, DOR, PFS, OS	T
NCT05041972 (ACE-Pan Tumor02)	II	ADC	HER2 AMP, OE	ARX788	no	pre-treated	all tumours (cohort1: NSCLC)	NA	NA	W°
ADC plus combinations										
NCT03334617	I umbrella	ADC + ICI	HER2 MUT, OE	TDXd	TDXd + durvalumab	2L	NSCLC, n=527	ORR	DCR, DOR, PFS, OS	A, NR[§]
NCT04042701 (U106)	IB	ADC + ICI	HER2 MUT, OE	TDXd	TDXd + pembrolizumab	1L	BC and NSCLC, n=115	MDT, DLT, RP2D, ORR	Cmax, TEAEs, DOR, DCR, PFS, OS	A, NR[§]
NCT04686305 (DESTINY-Lung03)	I	ADC + ICI +/-CT	HER2 OE	TDXd	TDXd + durvalumab +/-platin*/pemetrexed	pretreated (part1)	NSCLC, n=244	Satefy (AEs, SAEs)	ORR, DCR, DoR, PFS, OS, PK	R
		ADC + ICI +/-CT	HER2 OE	TDXd	TDXd + durvalumab +/-platin* /pemetrexed	naive (part2)				
		ADC+ bsAB (anti-PD1, anti-CTL4)	HER2 OE	TDXd	TDXd + volrustoming (+carboplatin)	naive-part3 (3B)				
		ADC+ bsAB (anti-PD1, anti-TIGIT)	HER2 OE	TDXd	TDXd + Rilvegostomig (+carboplatin)	naive-part4 (4B)				
NCT05048797 (DESTINY-Lung04)	III	ADC vs SOC	HER2 MUT (exon 19-20)	TDXd	TDXd vs platinun/pemetrexed/pembrolizumab	1L	NSCLC, n=450	PFS by BICR	OS, PFS inv, ORR, DCR, DoR, CNS-PFS, PFS2	R
NCT06899126 (DESTINY-Lung06)	III	ADC+ICI vs SOC	HER2 OE (and PDL<50%)	TDXd	TDXd+pembrolizumab vs platinun/pemetrexed/pembrolizumab	1L	NSCLC, n=686	PFS by BICR	OS	NR
NCT04460456	I	ADC +antiPD1	HER2 OE (2+ or 3+)	SBT6050	SBT6050 +pembrolizumab or cemiplimab	pre-treated	NSCLC, n=58	DLT, AEs	ORR, DoR, DCR, PK	U
NCT05745740	I/II	ADC+TKI	HER2 (exon20 ins)	RC 48 (Disitamab Vedotin)	RC 48+ pyrotinib	pre-treated	NSCLC, n=26	MTD, DLT	ORR, DCR, DoR, PFS, OS	NR

					(called KBP-5209)					
NCT06185400	II	ADC+TKI	HER2 MUT	RC 48 (Disitamab Vedotin)	RC 48+ pyrotinib	pre-treated	NSCLC, n=108	ORR	DCR, PFS, OS	NR
NCT06734182 (NEOVISION)	II	ADC + anti-PD1 +CT	HER2 MUT	RC 48 (Disitamab Vedotin)	RC 48+Envafolimab +carboplatin	neadjuvantt (stage II, III)	NSCLC, n=25	MPR	pCR, ORR, EFS, OS, safety	R
NCT06749860	II	ADC + anti-PD1 + anti-VGFA	HER2 MUT, AMP, OE	RC 48 (Disitamab Vedotin)	RC48 +Tislelizumab + bevacizumab	pretreated	NSCLC, n=58	ORR	DCR, DOR, PFS, OS	R
NCT05847764	II	ADC + anti-PD1 + CT	HER2 MUT, AMP, OE	RC 48 (Disitamab Vedotin)	RC48 +Tislelizumab /carboplatin (arm1)	naive	NSCLC, n=95	ORR	DOR, DCR, PFS, OS	NR
		ADC+TKI	EGFR plus HER2 alteration	RC 48 (Disitamab Vedotin)	RC48 +Furmonertinib (1L) (arm 2)	naive				
		ADC+TKI	HER2 MUT, AMP, OE	RC 48 (Disitamab Vedotin)	RC48 +Furmonertinib (2L) (arm3)	pre-treated				
NCT05482568	I/II	ADC+TKI or antiPL1	HER2 MUT	SHR-A1811 (trastuzumab rezutecan)	SHR-A1811+ pyrotinib or SHR-1316 (adrelimab)	pre-treated	NSCLC, n=324	DLT, AEs, SAEs, ORR	PK, DOR, PFS	R
NCT05091528	I/II	ADC + TKI+ CT	HER2/TLR8 agonist	SBT6050	SBT6050 +TDXd (or + Tucatinib +/- capecitabin)	pre-treated	all tumours (NSCLC) n=2 enrolled	DLT,, TEAEs	SAEs, ORR, DCR	T[£]
bsAB										
NCT02892123 (ZWI-ZW25-101)	I (part2)	bsAb	HER2 (ECD4/ECD2) OE, AMP	ZW25 (Zanidatamab)	no	pre-treated	all tumours (included NSCLC), n= 86	AEs, DLT	ORR, PFS	C
NCT06695845	II	bsAb	HER2 OE (3+)	ZW25 (Zanidatamab)	no	pre-treated	all tumours (NSCLC), n=200	ORR by BICR	ORR inv, DCR, DOR, TTR, PFS, OS	R
bsADC										
NCT02829372	I	bsADC	HER2/CD3	GBR 1302	no	pret-reated	all tumours (included NSCLC), n= 36	DLT, MDT, AEs	ORR, DCR, PK	T[ç]
NCT03821233	I	bsADC	HER expressing	ZW49	no	pre-treated	all tumours (included NSCLC), n=112	DLT, AEs	ADA, ORR, DCR, PFS; OS	C
NCT02912949	II	bsADC	NRG1 fusion (not HER2/HER3)	Zenocutuzumab (MCLA-128)	no	pre-treated	all tumours (group F: NSCLC) n=250	ORR inv, DoR	ORR by BICR, CBD, TTR, PFs, OS	R
**Other drugs**										
NCT05681780	I	CD40L-Augmented TIL (immunotherapy)	HER2 MUT (and other drivers)	TIL	TIL+ Nivolumab	pre-treated	NSCLC, n=20	Safety (AEs)	ORR, DoR, OS	R
NCT00228358	I	HER-2/neu vaccine	HER-2 OE	HER-2/neu vaccine	cyclophosphamide or denileukin diftitox	pre-treated	NSCLC, n=8	feasibility and safety	NA	C
NCT04143711	I/II	Tri-specific, NK cell Engager Therapy (TriNKET)	HER2 MUT, AMP, OE	DF1001 is a first in class	no	pre-treated	all tumours (NSCLC), n=378	Safety	PK, OS, ORR, DoR, PFS	R

NCT04660929	I	CAR-macrophages	HER OE	anti-HER2 CAR macrophages	no	pre-treated	all tumours (NSCLC), n=48	Safety. Tolerability and feasibility	ORR, PFS	A, NR
NCT04319757	I	anti-HER2 NK cells	HER2 OE	ACE1702	no	pre-treated	all tumours (NSCLC), n=12	DLT, MTD	immune function	C
NCT00003002	I	HER-2/ Neu Vaccine	HER2 OE	HER-2/ Neu Vaccine Plus GM-CSF	HER-2/ Neu Vaccine Plus GM-CSF	pre-treated	all tumours (NSCLC), n=60	Safety	NA	C
NCT04278144	I/II	ISAC	HER2 expression or AMP	BDC-1001 +/- nivolumab	BDC-1001 +/- nivolumab	pretreated	all tumours (NSCLC), n=175	Safety (AEs; DLT, MTD)	ORR, PK, DoR, PFS	T[&]

Legend: *cis- or carboplatin; **for unacceptable toxicity during the dose-escalation portion (phase 1) of the study and did not progress to phase 2; [&]discontinued by sponsor; °for business strategy change; [£]Sponsor decision based on strategic re-alignment; ç Study halted prematurely and will not resume; participants are no longer being examined or receiving intervention; §result published. **Abbreviation:** SOC: standard of care; ORR: overall response rate; DCR: disease control rate; DOR: duration of response; PFS: progression-free survival; OS: overall survival; mos = months; PK: pharmacokinetics; RP2D: recommended phase 2 dose; MTD: maximum tolerated dose; TEAEs: treatment emergent adverse events; TTR: time to response; DLT: dose limiting toxicities; QoL: quality of life; TKI: tyrosine kinase inhibitor; dTKI: dual TKI (EGFR/HER2 TKI); sTKI: selective HER2 TKI; ICI: immune checkpoint inhibitor; NGS: next generation sequencing; PCR: protein chain reaction; GM-CSF: Colony-stimulating factors; MPR: Major Pathological Response, ADA: Incidence of anti-drug antibody; CBR: clinical benefit rate; BP: brain penetrant; ISH: in situ hybridization; IMA: immunomodulatory agent; ISAC: Immune Stimulating Antibody Conjugate ; BICR: Blinded Independent Central Review; AUC: Area under the concentration; Cmax: Maximum observed drug concentration; TIL: Tumor-infiltrating Lymphocytes; icPFS: intra-cranial progression-free-survival; RANO: Response Assessment in Neuro-Oncology; rwPFS: real word progression free survival; TTD: time to treatment discontinuation; R: recruiting; NR: not recruiting; A,NR: active, not recruiting; C: completed; T: terminated; U: unknown status; W: Withdrawn. Source: https://www.clinicaltrials.gov/search?cond=Non-small%20Cell%20Lung%20Cancer&term=HER2 (last access: 4 may 2025).
